# Functional variation in allelic methylomes underscores a strong genetic contribution and reveals novel epigenetic alterations in the human epigenome

**DOI:** 10.1186/s13059-017-1173-7

**Published:** 2017-03-10

**Authors:** Warren A. Cheung, Xiaojian Shao, Andréanne Morin, Valérie Siroux, Tony Kwan, Bing Ge, Dylan Aïssi, Lu Chen, Louella Vasquez, Fiona Allum, Frédéric Guénard, Emmanuelle Bouzigon, Marie-Michelle Simon, Elodie Boulier, Adriana Redensek, Stephen Watt, Avik Datta, Laura Clarke, Paul Flicek, Daniel Mead, Dirk S. Paul, Stephan Beck, Guillaume Bourque, Mark Lathrop, André Tchernof, Marie-Claude Vohl, Florence Demenais, Isabelle Pin, Kate Downes, Hendrick G. Stunnenberg, Nicole Soranzo, Tomi Pastinen, Elin Grundberg

**Affiliations:** 1 Department of Human Genetics, McGill University, Montreal, Quebec Canada; 2 McGill University and Genome Quebec Innovation Centre, Montreal, Quebec Canada; 3 Team of Environmental Epidemiology applied to Reproduction and Respiratory Health, Inserm U1209, CNRS, University Grenoble Alpes, Institute for Advanced Biosciences, Grenoble, France; 4 Department of Human Genetics, The Wellcome Trust Sanger Institute, Wellcome Trust Genome Campus, Hinxton, Cambridge, CB10 1HH UK; 5 Department of Haematology, University of Cambridge, Cambridge Biomedical Campus, Long Road, Cambridge, CB2 0PT UK; 6 Institute of Nutrition and Functional Foods (INAF), Laval University, Québec, QC G1V 0A6 Canada; 7 Genetic Variation and Human Diseases Unit, UMR-946, INSERM, Université Paris Diderot, Université Sorbonne Paris Cité, Paris, France; 8 European Molecular Biology Laboratory, European Bioinformatics Institute, Wellcome Genome Campus, Hinxton, Cambridge, CB10 1SD UK; 9 UCL Cancer Institute, University College London, 72 Huntley Street, London, WC1E 6BT UK; 10 Cardiovascular Epidemiology Unit, Department of Public Health and Primary Care, University of Cambridge, Strangeways Research Laboratory, Worts Causeway, Cambridge, CB1 8RN UK; 11 Québec Heart and Lung Institute, Laval University, Québec, QC G1V 4G5 Canada; 12 Pédiatrie, Centre Hospitalier Universitaire (CHU) Grenoble Alpes, Grenoble, France; 13 National Health Service (NHS) Blood and Transplant, Cambridge Biomedical Campus, Long Road, Cambridge, CB2 0PT UK; 14 Faculty of Science, Department of Molecular Biology, Radboud University, Nijmegen, 6525GA The Netherlands; 15 British Heart Foundation Centre of Excellence, Division of Cardiovascular Medicine, Addenbrooke’s Hospital, Hills Road, Cambridge, CB2 0QQ UK; 16 The National Institute for Health Research Blood and Transplant Unit (NIHR BTRU) in Donor Health and Genomics, University of Cambridge, Strangeways Research Laboratory, Wort’s Causeway, Cambridge, CB1 8RN UK

## Abstract

**Background:**

The functional impact of genetic variation has been extensively surveyed, revealing that genetic changes correlated to phenotypes lie mostly in non-coding genomic regions. Studies have linked allele-specific genetic changes to gene expression, DNA methylation, and histone marks but these investigations have only been carried out in a limited set of samples.

**Results:**

We describe a large-scale coordinated study of allelic and non-allelic effects on DNA methylation, histone mark deposition, and gene expression, detecting the interrelations between epigenetic and functional features at unprecedented resolution. We use information from whole genome and targeted bisulfite sequencing from 910 samples to perform genotype-dependent analyses of allele-specific methylation (ASM) and non-allelic methylation (mQTL). In addition, we introduce a novel genotype-independent test to detect methylation imbalance between chromosomes. Of the ~2.2 million CpGs tested for ASM, mQTL, and genotype-independent effects, we identify ~32% as being genetically regulated (ASM or mQTL) and ~14% as being putatively epigenetically regulated. We also show that epigenetically driven effects are strongly enriched in repressed regions and near transcription start sites, whereas the genetically regulated CpGs are enriched in enhancers. Known imprinted regions are enriched among epigenetically regulated loci, but we also observe several novel genomic regions (e.g., HOX genes) as being epigenetically regulated. Finally, we use our ASM datasets for functional interpretation of disease-associated loci and show the advantage of utilizing naïve T cells for understanding autoimmune diseases.

**Conclusions:**

Our rich catalogue of haploid methylomes across multiple tissues will allow validation of epigenome association studies and exploration of new biological models for allelic exclusion in the human genome.

**Electronic supplementary material:**

The online version of this article (doi:10.1186/s13059-017-1173-7) contains supplementary material, which is available to authorized users.

## Background

The classic first step of the central dogma of molecular biology, whereby information flows from the genome to the transcriptome before continuing to the proteome and the final phenotypic result, is nowadays bolstered and modified by an ever-increasing pool of epigenetic effects. Recent next-generation sequencing (NGS) approaches provide us with the opportunity to interrogate not only the genome and the transcriptome but also epigenetic layers using comparable technologies. Moreover, the use of single-base resolution sequencing allows us to distinguish individual-level genetic differences, giving us the additional ability to resolve differences between individual chromosomes, allowing an allelically resolved view of epigenetic modifications and gene expression linked through personal genetics.

Understanding the functional non-coding variation underlining complex disease has been one of the key challenges in the past years. Genome-wide association studies (GWAS) revealed that the majority of the associated single nucleotide polymorphisms (SNPs) lie in non-coding regulatory regions [1, 2]. To understand the functional impact of these SNPs, various studies have linked these to cellular traits, including gene expression (expression quantitative trait loci (eQTLs) or allele-specific expression (ASE) and splicing QTLs) [3–6], DNA methylation (mQTLs) [7, 8], histone marks (hQTLs), or allele specific chromatin immunoprecipitation (AS-ChIP)) [9, 10] effects, especially when cell types relevant to the disease of interest are used. Many of these studies have confirmed early efforts [1] showing that in fact a majority of GWAS hits are enriched for these different QTLs.

Allele specific methylation (ASM), where one allele exhibits a different methylation pattern compared to the other, has been observed in imprinted genes as well as in the female sex chromosomes through X-inactivation. More recently, ASM was found to be prevalent across the genome [11–13] with the majority of events being *cis*-regulated [14]. Also, ASM appears to have a role in the regulation of the ASE of autosomal non-imprinted genes. A large portion of ASE events are enriched within the vicinity of ASM events, where the hypomethylated allele matches the highly expressed allele [15, 16]. Allele-specific histone (ASH) has also been linked to allelically biased gene expression. Allele-specific enhancers are found close to genes showing ASE, with high concordance of ASH signal to the corresponding ASE of the same allele [9, 17]. Allele-specific independent events (methylation, gene expression and histone) have been shown to have genome-wide, autosomal associations for complex traits, and particularly for complex disease [18, 19]. To date, however, the parallel investigation of ASM, ASH and ASE has only been carried out in a limited set of samples.

To address this, we have performed the first comprehensive survey of the relationship between multiple epigenetic layers and the functional transcriptome, evaluating 1446 NGS (RNA-Seq, ChIP-Seq, whole genome bisulfite sequencing (WGBS), targeted bisulfite sequencing (methylC-capture sequencing; MCC-Seq)) data sets from 910 samples (freshly isolated primary cells or cryopreserved tissues) for allelic and non-allelic effects of global DNA methylation. We linked ASM, ASE, and ASH effects, and directly compared these to non-allelic effects, which allowed us to establish allelic coordination of the layers of epigenetics with the transcribed phenotype.

## Results

### Allelic and non-allelic patterns of the global DNA methylation landscape

**Fig. 1:**
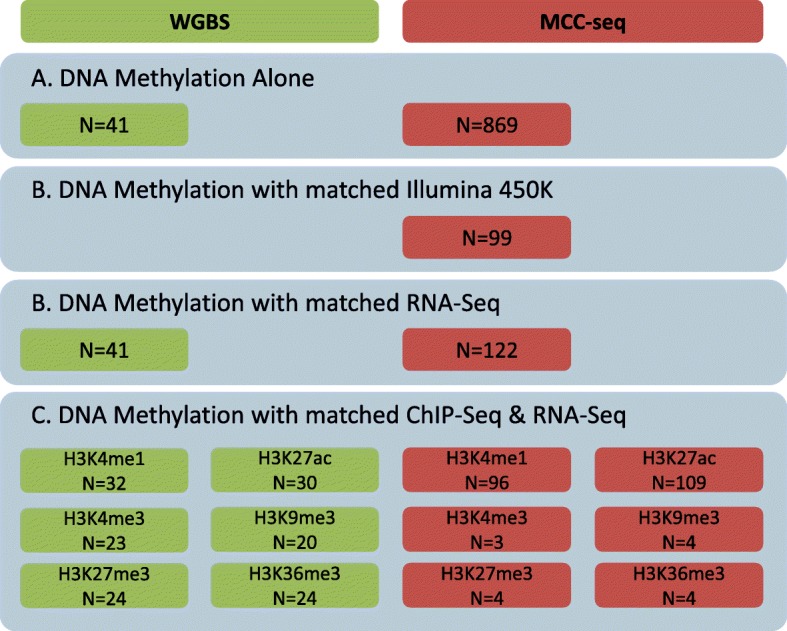
Samples having multiple layer of epigenetics profiles were used in this project. DNA methylation profiles were assessed for all samples using either whole genome bisulfite sequencing (*WGBS*; *green*) or targeted bisulfite sequencing (*MCC-Seq*; *red*). Listed in this figure are the number of samples used for analyses focusing on: **a** methylation sequencing alone (Methyl-Seq), **b** methylation with matched RNA-Seq from the same sample, or **c** methylation sequencing with matched ChIP-Seq (using six different histone marks) and matched RNA-Seq from the same sample

In order to characterize allelic and non-allelic effects of DNA methylation at CpGs genome-wide, we utilized phased NGS data generated from 910 samples derived from whole blood, adipose tissue, muscle, and purified monocytes and T cells (Fig. 1a). Specifically, we used our recently introduced MCC-Seq approach to enrich for non-coding regulatory sequences [20, 21], thus permitting high-resolution assessment of functional methylomes (Table 1; Additional file 1).

**Table 1:** Summary of targeted bisulfite-sequencing methylome capture panel design

	Total CpGs	Total regions	Total size (bp)
Regulatory regions in immune cells (DNaseI hypersensitive/active chromatin)	1,837,099	315,043	48,810,975
Hypomethylated footprints from immune cells (MethylSeekR)	3,539,071	477,795	86,414,084
Illumina 450 K methylation assay included regions	1,934,175	328,405	40,853,151
Autoimmune SNPs from GWAS catalog	14,572	7273	678,026
Unique (non-overlapping content) in custom MCC-Seq capture panel	4,609,564	822,884	119,089,296

We distinguish between genotype-dependent tests—allele-specific methylation (ASM) and non-allelic methylation quantitative trait locus (mQTL) analysis—and a novel genotype-independent test (GIT) for the identification of genetically and epigenetically regulated DNA methylation loci. In our ASM pipeline, phased methylation measurements at single CpG resolution are used together with a global test to compare the methylation sequencing reads for one allele against those for the other allele. This allows us to leverage the power of all the methylation reads across samples at the allelic level to test for differences in methylation rate of the reads between the two alleles. For the mQTL analysis, we assess the *cis*-association (250-kb window surrounding the CpG) between SNP genotypes and bi-allelic methylation levels across individuals as previously described [7, 8]. Finally, we use GIT to detect methylation imbalance between chromosomes using phased methylation measurements. This approach separates the allelic methylation for each sample into reads for the high methylated allele and the low methylated allele, then considers all the reads for the high methylated alleles together against all reads for the low methylated alleles. GIT allows us to detect differences in methylation between the two alleles regardless of the genetics of the underlying chromosome, permitting us for the first time to interrogate phased methylation for putative epigenetically driven effects.

**Fig. 2:**
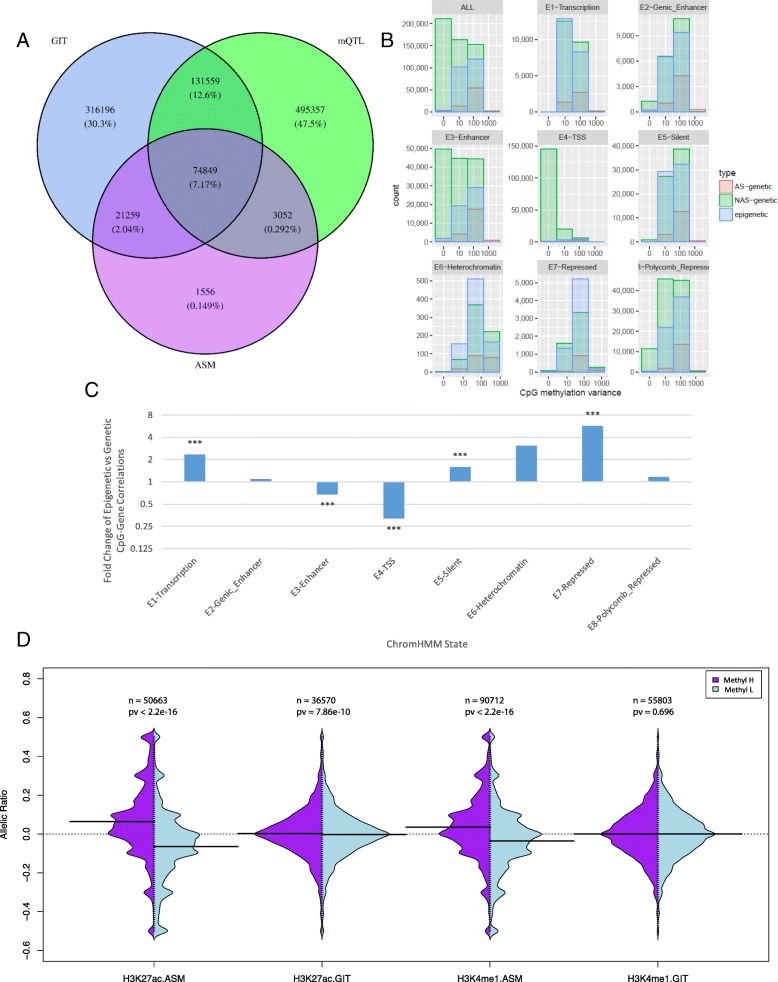
**a** CpGs showing significant (*q* < 0.01) imbalanced methylation, significant (*q* < 0.1) allelic methylation, and significant (*q* < 0.1) non-allelic methylation. Percentages indicate the proportion of the total significant CpGs found in any of these three sets (n = 962,557 of the 2,233,846 CpGs tested in all three tests). **b** Counts of CpGs showing allele-specific (*AS*)-genetic (ASM *q* < 0.1), non-allele-specific (*NAS*)-genetic (ASM *q* ≥ 0.1 and mQTL < 0.1), epigenetic (GIT *q* < 0.01, ASM and mQTL ≥ 0.1), and no (GIT *q* > 0.01, ASM and mQTL q ≥ 0.1) associations. Frequencies are plotted for all the CpGs, and also for CpGs in each of the ChromHMM regions. *TSS* transcription start site. **c** Fold change of the rate of putatively epigenetic (GIT *q* < 0.01, ASM and mQTL *q* ≥ 0.1) versus genetic (ASM or mQTL q < 0.1). ***Ratios with Fisher *p* < 0.0001; all other *p* values were >0.05. **d** Distribution of allelic ratios at significant GIT and ASM CpGs, for H3K27ac and H3K4me1 in normal T cells

We focused on the ~2.2 M CpGs that were tested using ASM and mQTL analysis and GIT in any of our datasets and identified a total of 1,043,828 CpGs that were genetically or epigenetically regulated (Fig. 2). Of these, 69.7% showed either significant (*q* < 0.1) ASM (2.2%) or mQTL (60.1%) or both (7.5%), while the remainder (30.3%) showed potentially significant (*q* < 0.01) allelic imbalanced methylation (GIT) without genetic basis, i.e., that was epigenetically driven (Fig. 2a; Additional file 2). We noted 543,863 of the regulated CpGs were significant in the GIT, which identified proximal and distal genetic effects (ASM and mQTL) in addition to putative epigenetic effects. In addition, 29% of CpGs significant in the mQTL analysis were also significant in the GIT, with 38% of GIT-significant CpGs also being significant mQTLs. However, despite this substantial overlap, over half of the mQTLs do not have a detectable allelic component. In contrast, 74% of the significant ASM CpGs were also significant mQTLs. When restricting to CpGs and SNPs tested by both mQTL and ASM analyses, we note that the proportion of significant allelic CpG–SNP pairs replicated in the mQTL analysis remains comparable at 65%, but the significant GIT pairs replicated in the ASM analysis is reduced to 17.8% (Table 2); 31% of the GIT CpGs have a significant genetic effect that is not from the SNP used for phasing, potentially indicating more distal regulatory genetic effects of DNA methylation.

**Table 2:** Summary of CpG phasing SNP overlaps between ASM, GIT, and mQTL

Test A	Significant (test A)	Test B	Significant (test B)	Significant (test A and B)	Total (test A and B)
ASM	108,515	mQTL	164,671	70,700	2,319,084
GIT	818,954	ASM	158,444	146,012	4,462,724
GIT	603,033	mQTL	164,671	102,663	2,319,084

Of the top 500 CpGs (by corrected GIT *q* value) deemed to be epigenetically controlled (ASM and mQTL *q* ≥ 0.1), 291 (58.2%) CpGs formed clusters of three or more CpGs within 2 kb. In total, these formed 48 clusters across the genome—23 (47.9%) occurring at known or presumed imprinted loci, six (12.5%) in the *PCDH* gene cluster (reported to be subject to random monoallelic regulation [22, 23]), and five (13.5%) at potentially novel imprinted/random monoallelic loci (*CTDP1*, *DIAPH3*, *GLS2*, *ITGB1*, and *ZNF714*). CpG clusters near known imprinted loci were 124-fold enriched compared to random expectation (GREAT analysis [24], *p* = 1.79 × 10^−20^). This demonstrates that sites identified by GIT specifically covered a large fraction of genetic-independent allelic methylation in the human genome. We further generated a list of high-confidence non-genetic CpG clusters by examining windows of at least 15 consecutive CpGs and selecting windows where all CpGs did not show significant genetic methylation (ASM and mQTL *q* ≥ 0.1) but showed significant imbalanced allelic methylation (GIT *q* < 1 × 10^−5^), and where the median imbalanced allelic methylation was highly significant (log_e_(*q*) < −10) (Additional file 3). As expected, these regions were enriched for maternal and genetic imprinting (*p* = 1.65 × 10^−6^ and p = 2.79 × 10^−5^ at 5.4- and 3.6-fold enrichment in mouse phenotype terms) as well as developmental process terms (17 of the 20 significant Gene Ontology (GO) biological process terms). In addition, the developmental processes appear to be driven by developmental regulatory transcription factors (transcription regulatory region sequence-specific DNA binding *p* = 3.65 × 10^−8^, GO molecular process) with specific 5′ enrichment of imbalanced methylation (enrichment of HOXL, NKL, and Cadherin gene families via Interpro and HGNC gene family GREAT analysis; Additional file 4).

Next, we explored epigenetic and genetic allelic methylation variation in different genomic contexts, via overall population methylation variation in T cells. Focusing on T cells allows us to limit the effect of tissue heterogeneity on overall methylation variation. We inferred genomic contexts using states generated from available histone mark data using ChromHMM [25] (see “Methods”). Direct (ASM) and indirect effects (mQTLs) account for a large fraction of total methylation variation; with 28% of the top 57% most variable sites (methylation variance >10) explained by one of the allelic methylation variation classes (Fig. 2b). In fact, at highest total methylation variance (methylation variance >500), essentially all methylation variation (98%) shows an allelic basis via significant ASM. In the case of variable methylation in promoter states, the mQTL approach models a large proportion of the variability, which is expected given their overall hypomethylated status and hence lower potential for detection of significant methylation differences between alleles. On the other hand, genetic ASM is greatly enriched among extremely variable CpGs, which is likely due to direct strong local influence of sequence differences altering methylation efficiency [26]. Non-genetic allelic variation accounts for 21% of highly variable CpGs (methylation variance >10) with a tendency to explain a higher fraction in repressed states, suggesting that overall methylation level variation within these chromatin states arises stochastically in one or the other allele. These analyses reveal the dichotomous nature of methylation variation with the relative enrichment of sequence-dependent variation in canonical regulatory elements (Fig. 2c, d), and non-genetic variation enriched in larger repressed or transcriptional annotations.

### Cell-type specific DNA methylation events

**Fig. 3:**
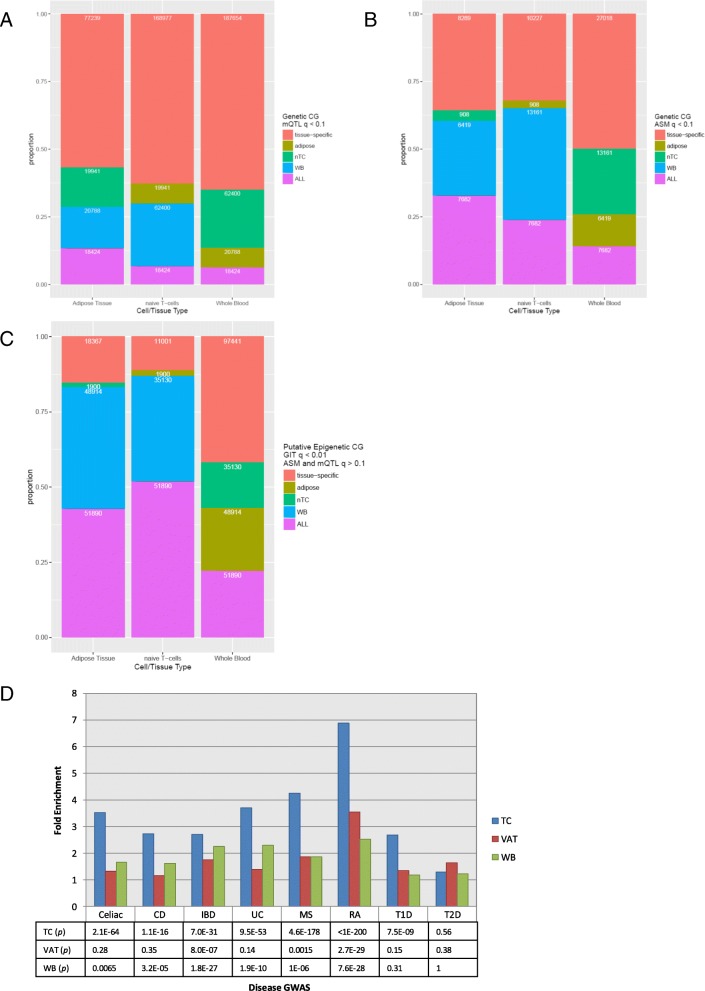
The proportion and number of sites of cell type-specific methylation in adipose tissue, naïve T cells (*nTC*), and whole blood (*WB*). The *red segments* at the *top* show the proportion of CpGs that are specific to the specific tissue, and the *purple segments* at the *bottom* show the proportion of CpGs that were found in all three cell types. The *yellow*, *green* and *blue bars* show CpGs that are shared between the specific cell type and adipose tissue, naïve T cells, and whole blood, respectively. Shown is the breakdown in the three cell types for **a** significant mQTL CpGs (*q* < 0.1), **b** significant ASM CpGs (*q* < 0.1), **c** putative epigenetic (filtered for ASM and mQTL *q* > 0.1) GIT CpGs (GIT *q* < 0.01). **d** Enrichment GWAS SNPs associated with significant ASM in three different tissues—naïve T cells (*TC*, *blue*), whole blood (*WB*, *green*) and visceral adipose tissue (*VAT*, *red*). We show enrichment for disease-associated loci from eight different traits (celiac disease, Crohn’s disease (*CD*), inflammatory bowel disease (*IBD*), ulcerative colitis (*UC*), multiple sclerosis (*MS*), rheumatoid arthritis (*RA*), type 1 diabetes (*T1D*), and type 2 diabetes (*T2D*)) and SNPs associated with ASM

We also identified cell-type specific CpG methylation events by comparing genetic and epigenetic CpG methylation that is significant in one or more of our three cell-types—adipose tissue, naïve T cells, and whole blood (Table 3, Fig. 3). We compared the CpGs that were tested in all three cell types and identified the CpGs that are uniquely significant in the cell type (tissue-specific), significant in all the cell types, and significant in two of the cell types. As expected, we see the largest number of tissue-specific sites in whole blood, which is our tissue set with the largest number of samples and therefore our most deeply interrogated tissue type. For mQTLs, we observe over half of the CpGs are tissue-specific, with naïve T cells sharing over twice as many CpGs with whole blood compared to adipose (Fig. 3a). For allele-specific genetic effects, we see a slightly weaker tissue-specific effect, especially in adipose tissue. However, over a quarter of the ASM CpGs are still clearly identified as tissue-specific (Fig. 3b). The putative epigenetic CpGs show slightly more reduction but we still see almost an eighth of the identified CpGs acting in a tissue-specific manner. Moreover, over 50,000 of the putative epigenetic CpGs were replicated in all three cell types, replicating the genotype-independent methylation difference between alleles in multiple cell type contexts (Fig. 3c).

**Table 3:** Tissue-specific number of CpGs tested and number of significant CpGs for the mQTL, ASM, and GIT analyses

Cell type	Naïve T cells	Visceral adipose tissue	Whole blood	Common
All mQTLs tested	3,140,791	1,959,622	4,261,030	1,602,686
Significant mQTLs (*q* < 0.1)	501,606	170,155	769,853	555,423
All ASM tested	3,109,121	1,713,482	1,636,884	1,079,807
Significant ASM (*q* < 0.1)	60,559	38,827	81,126	7,682
All GIT tested	2,944,290	1,714,250	1,076,251	622,721
Significant GIT (*q* < 0.01)	278,516	301,517	486,201	88,599

Next, we took advantage of our rich ASM effects detected in multiple tissues (i.e., naïve T cells, whole blood, and visceral adipose tissue) to assess the overlap between disease-associated loci from eight different traits: celiac disease [27], Crohn’s disease (CD) [28], inflammatory bowel disease (IBD) [28], ulcerative colitis (UC) [28], multiple sclerosis (MS) [29], rheumatoid arthritis (RA) [30], type 1 diabetes (T1D) [31], and type 2 diabetes (T2D) [32] (Fig. 3d). We tested for enrichment of marginally associated disease SNPs (*p* < 10^-5^) for significant ASM (*q* < 0.1) adjusting for linkage disequilibrium (LD) structure (r^2^ < 0.1). We observed strong enrichment (three- to sevenfold) of autoimmune associations for ASM in naïve T cells whereas only moderate enrichment was observed for ASM in whole blood and even weaker enrichment for adipose ASM. However, there was a suggestive enrichment (1.6-fold) of T2D loci for ASM in adipose tissue, which all together suggest evidence for cell type specificity in functional interpretation of disease loci.

### Allelic and non-allelic patterns of non-CpG methylation

Recent studies have shown evidence of non-CpG (or CpH) methylation in multiple human tissues [15]. However, we recently showed that CpH methylation might partly be driven by potentially “erroneous/nonspecific” methylation from the methylation machinery at neighboring CpGs [33]. To study this further as well as to characterize allelic and non-allelic effects of CpH methylation events at the genome-wide level we extended our analysis to include CpH sites. We restricted this to sites interrogated in at least 50 individuals from our deepest covered dataset, adipose tissue. First, we confirmed our earlier results that most CpH contexts show complete unmethylation in adipose tissue [33]; therefore, we restricted the data set to CpH sites where at least 25 individuals have methylation greater than zero (N = 189,891 CpHs). Next, we filtered this set further to exclude CpHs overlapping a SNP (dbSNP146) at the dinucleotide position (e.g., to avoid an adjacent SNP creating a CpG context) and then repeated our ASM and GIT analyses on these CpHs (N = 49,172) using the same strategy as applied to CpGs. We found that 1627 (3.3%) were significantly associated with an ASM event (*q* < 0.1), with a slightly smaller proportion (2.96%) showing potentially significant allelic imbalanced methylation (GIT, *q* < 0.01) without genetic basis. These epigenetically driven effects on CpH methylation are significantly smaller (sixfold) than for CpG (Table 3) methylation and may indicate less dynamic influences on CpH methylation variation due to an overall static CpH pattern in differentiated cells. Overall, true allelic methylation in a CpH context remains an extremely rare event, as observed in non-allelic CpH methylation variation studies [33].

### Validation of NGS-based genotype-dependent tests

In an attempt to validate our NGS-based genotype-dependent tests (i.e., ASM and mQTL), we performed a number of analyses by comparing results from one of the cohorts (naïve T cells) with estimates by an independent non-NGS based approach—Illumina Infinium HumanMethylation450 BeadChip (Illumina 450 k array). To rule out underlying allelic biases in NGS approaches, we validated our ASM results by comparing our aligned sequencing results against matched methylation from the Illumina 450 K array for the same samples (Fig. 1b) [34]. First, we fetched all CpGs tested in the ASM pipeline that were also covered on the 450 K array and confirmed the usual pattern of predominantly hypo- and hypermethylation (Additional file 5: Figure S1a), as well as a strong correlation (R = 0.97) between the expected methylation rates (unweighted average of the allelic methylation) called from these sites by MCC-Seq and the rates estimated by the Illumina 450 k array (Additional file 5: Figure S1b). Second, by restricting to CpGs showing significant ASM (q < 0.1), we noticed a marked shift from the usual, expected hypo- and hypermethylation towards hemi-methylation (Additional file 5: Figure S1a) using the Illumina 450 K array. However, the expected (combined) methylation called from allelic sequencing at these sites remains highly correlated to the methylation measured via the array (Additional file 5: Figure S1c; R = 0.92). Taken together, these results indicate the high accuracy of our detected ASM events with no clear evidence of the presence of technical artifacts in calling allelic data using NGS approaches. Next, we focused on our mQTLs identified by MCC-Seq significant at *q* < 0.01 and *q* < 0.001 (Additional file 5: Figure S1) and fetched overlapping SNPs and CpG information from the Illumina 450 K array for the same samples [34]. Here, we note that as much as 53.8% (for *q* < 0.01) and 72.0% (for *q* < 0.001) of the CpGs lead SNP mQTLs from the MCC-Seq replicate in the Illumina 450 K data for the same SNP–CpG combination (*q* < 0.1), confirming the robustness of mQTL discovery by MCC-Seq.

### Allelic analysis to link methylation and gene expression

Next, we contrasted correlations between methylation and gene expression between the methylome and the transcriptome in both allele-specific (AS) and non-allele-specific (NAS) contexts as a proxy for functional outcomes of methylome variation.

We limited the analysis to individuals where both gene expression and DNA methylation were available, corresponding to 41 paired RNA-Seq and WGBS samples, and 122 paired RNA-Seq and MCC-Seq samples (Fig. 1c; “Methods”; Additional files 1 and 6). In NAS tests, we correlated total gene expression against total methylation and compared to AS tests, where allelic methylation was correlated against allelic gene expression estimates. Due to the larger number of samples and coverage available via MCC-Seq, we were able to test a larger number of CpGs and thus perform more CpG–gene expression tests, which allowed us to identify more weakly correlated relationships between methylation and gene expression compared to WGBS.

**Fig. 4:**
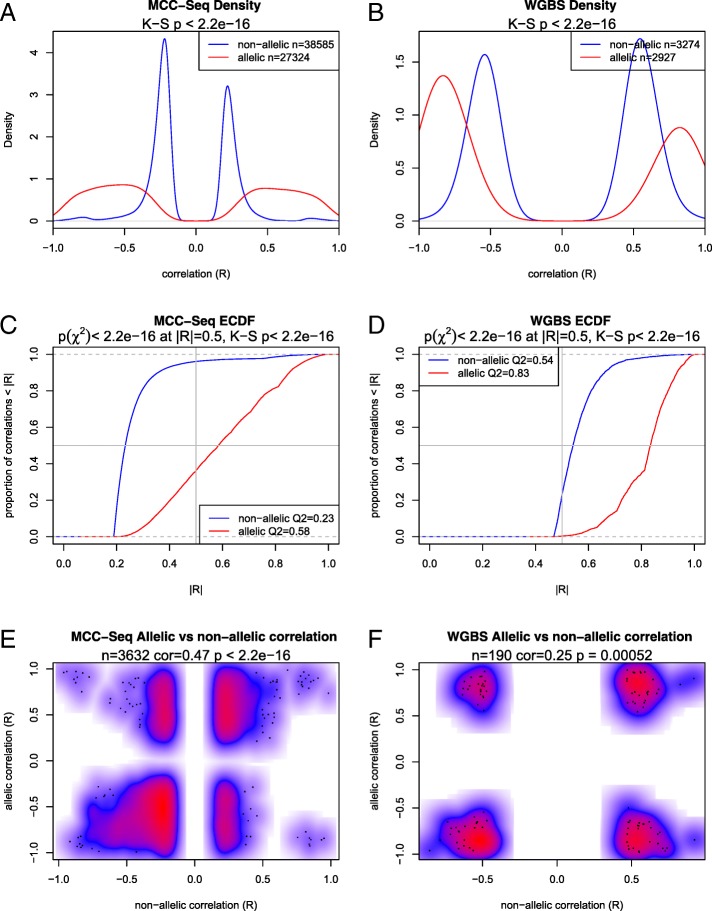
Density of allelic and non-allelic methylation versus gene expression correlation (R) and cumulative distribution of the absolute allelic and non-allelic correlation (|R|) for each dataset. **a** Density plot of significant (*p* < 0.05) correlations detected among sites (N_CpG_ = 241,687, N_tests_ = 441,931) tested for allelic and non-allelic correlation for CpGs measured via MCC-Seq. **b** Density plot of significant correlations for sites (N_CpG_ = 40,315, N_tests_ = 58,106) with methylation estimated by WGBS. **c** Empirical cumulative density function (*ECDF*) plot of the absolute correlation for sites evaluated using MCC-Seq. **d** ECDF for sites evaluated by WGBS. **e** Smoothed color density scatter plot of sites comparing significant non-allelic (x-axis) and allelic (y-axis) correlation (*p* < 0.05 for both correlation tests) for MCC-Seq. *Red* indicates high density, *blue* indicates low density, and *white* indicates no data. **f** Smoothed color density scatter plot of significant non-allelic versus allelic correlations for WGBS

While the total number of nominally significant (*p* < 0.05) correlations detected was slightly lower in AS analyses (27,324 versus 38,585 in MCC-Seq, 2927 versus 3274 in WGBS), AS analyses detected a higher rate of strong correlations when compared with NAS correlations for both MCC-Seq (Fig. 4a) and WGBS (Fig. 4b) across our datasets (Kolmogorov–Smirnov *p* value < 2.2 × 10^−16^ for both WGBS and MCC-Seq). We observed significant differences in the magnitude of the detected correlations from AS analysis compared to NAS analysis, where the median NAS significant correlation was R = 0.23 and R = 0.54 for WGBS and MCC-Seq, respectively, compared to the median AS significant correlations of R = 0.58 and R = 0.83, respectively (chi-squared *p* value <2.2 × 10^−16^ at |R| = 0.5 in both cases). At all quantiles, the AS curve dominates the NAS curve, showing higher correlation values (Fig. 4c, d). We also assessed the overall concordance between AS and NAS methylation–expression correlations (Fig. 4e, f) and observed, across approaches, that both analyses have the same direction of effect for significant associations (R = 0.25, *p* = 0.0005 and R = 0.47, *p* < 2. 2 × 10^−16^ for WGBS and MCC-Seq, respectively). These effects were again pronounced in MCC-Seq comparisons, reflecting the larger number of relationships evaluated and the wider range of significant correlation values that could be compared.

### Allelic effects on genomic features

**Fig. 5:**
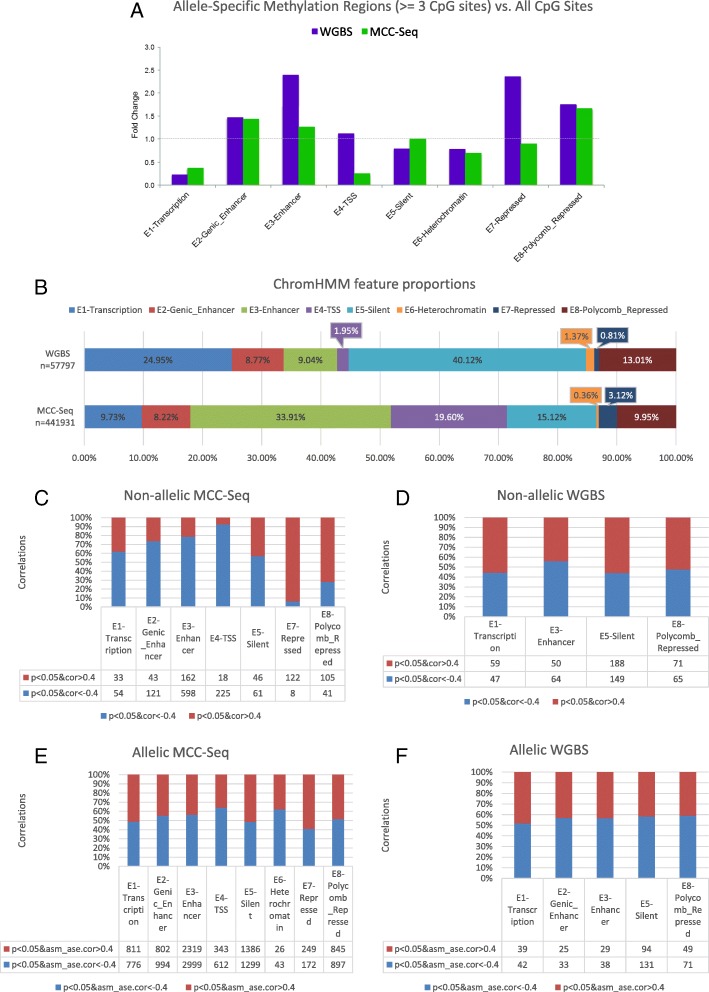
**a** Fold change difference in fraction of CpG regions (three or more consecutive significantly allelically differentially methylated CpGs) in ChromHMM-assigned state versus the fraction of single significantly allelically differentially methylated CpGs in the same ChromHMM state. The *x-axis* lists the eight ChromHMM states and each *colored bar* shows a different cell type/methylation interrogation technology. **b** Proportion of correlations tested in each ChromHMM state with CpG methylation sequenced by WGBS and MCC-Seq. **c** Fold-enrichment of positively correlated CpGs evaluated by MCC-Seq in each ChromHMM state. **d** Fold-enrichment of positive WGBS correlations. **e** Fold-enrichment of negatively correlated MCC-Seq CpGs per state. **f** Fold-enrichment of negatively correlated WGBS CpGs per state

We then sought to study the patterns of coordinated methylation, looking for enrichment of ASM regions in the context of genomic states. We observed that ASM regions (three or more ASM CpGs) occurred with higher frequency than expected in enhancer states (WGBS 2.39-fold, MCC-Seq 1.26-fold; Fisher *p* < 2. 2 × 10^−16^) when compared with all CpGs (Fig. 5a). States associated with transcription were depleted for ASM regions (WGBS 4.34-fold, MCC-Seq 2.63-fold; *p* < 2. 2 × 10^−16^) compared with all CpGs. These findings support the use of targeted interrogation of methylomes by MCC-Seq as it successfully diverts sequencing from functionally less active regions to regions with functional epigenetic activity. In fact, by contrasting the two methods (WGBS versus MCC-Seq) for methylome profiling, we noted that many of the evaluated CpG–gene expression relationships are skewed towards the silent state when using the WGBS method (40%), whereas MCC-Seq reduces this fraction to 15% (Fig. 5b). On the other hand, MCC-Seq interrogates more correlations at enhancer states—from 9% in WGBS to 34%—and transcription start site (TSS) states, where the fraction was increased from 2 to 20%. Overall, less than 4% of correlations involved CpGs in heterochromatin or repressed states for both MCC-Seq and WGBS, making these types of regions particularly difficult to characterize.

Next, we examined the enrichment of significant AS and NAS methylation (|R| > 0.4, *p* < 0.05) in each of these genomic features (Fig. 5c–f). The non-allelic analysis clearly shows enrichment of the significant negative correlations (R < -0.4, *p* < 0.05) in the TSS state and enhancers (Fig. 5c–d) confirming our earlier findings [7]. Positive correlations (R > 0.4, *p* < 0.05) were enriched in the repressed state. Similarly, when we look at significant allelic correlations (|R| > 0.4, *p* < 0.05) at CpGs where we have ASM (*p* < 0.05), we replicate the enrichment of negative correlations in TSS states and the positive correlations in repressed states (Fig. 5e, f).

In an attempt to disentangle the genetic effects underlying functional methylation variation (i.e., correlated with gene expression), we contrasted functional epigenetic methylation and functional genetic methylation. Of the 3066 AS CpG–gene correlations (*n* = 414,954 tested), where AS methylation was strongly correlated to AS gene expression (|R| > 0.4, *p* < 0.05), we observed that 65.7% are under genetic control (43.7% with significant ASM and a further 22% with significant mQTLs) and 25.8% link to putative epigenetic regulation (significant GIT but no significant ASM or mQTLs). The putatively epigenetic AS CpGs are strongly enriched (5.7-fold enrichment, *p* < 2.2 × 10^−16^) in repressed regions and in transcribed regions (2.3-fold, *p* = 3.2 × 10^−8^) when compared to the genetically regulated CpGs, which are enriched near the TSS (3.1-fold, *p* = 2.97 × 10^−16^) and enhancers (1.5-fold, *p* = 2.53 × 10^−14^) (Fig. 2c). In fact, 65.7% of correlations have an identified strong genetic methylation basis (in ASM or mQTLs), while a further 25.8% have imbalanced allelic methylation with no identified genetic basis.

### Linking allelic histone deposition, DNA methylation, and gene expression

Next we examined the effect of functional CpG methylation on histone marks, combining ASM and ASE with chromatin modification data from ChIP-Seq using an allele-specific histone (ASH) test to measure the allelic chromatin modification overlapping functional CpGs with allelic methylation (Fig. 1d, “Methods”).

When comparing genetic to putatively epigenetic allelic methylation, we observe that genetic allelic methylation (mQTL or ASM *q* < 0.1) is much more strongly linked to a concordant difference in histone mark deposition as measured by the ASH test using ChIP-Seq data. We note that the higher methylation allele at genetically regulated CpGs shows a lower rate of H3K4me1 and H3K27ac deposition (*p* < 2.2 × 10^−16^; Fig. 2d), whereas a substantially smaller difference in methylation is observed at the putative epigenetically regulated sites (GIT *q* < 0.01, mQTL and ASM *q* > 0.1).

**Fig. 6:**
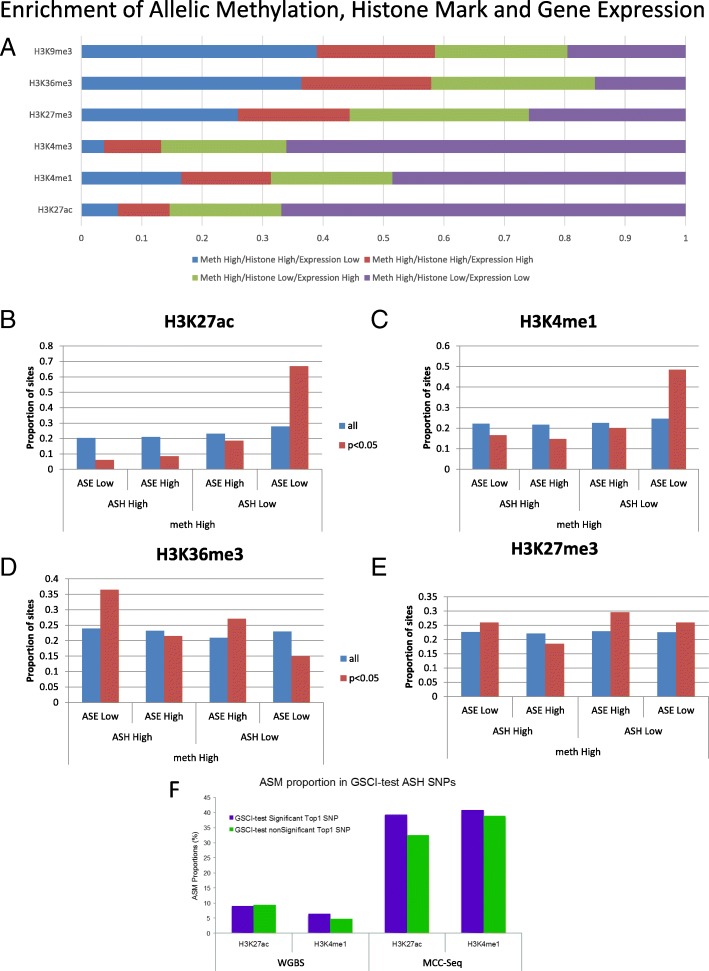
**a** Proportion of sites showing differential allelic methylation, histone occupancy, and gene expression. All four combinations of high and low methylation rate, histone occupancy rate, and gene expression are compared, described from the perspective of the high methylation allele, and whether this allele is the one with high or low histone occupancy, and high or low gene expression (note that high methylation allele with low histone occupancy also refers to the low methylation allele with high histone occupancy). For the histone marks **b** H3K27ac, **c** H3K4me1, **d** H3K36me3, and **e** H3K27me3, we show the proportions of all the differential allelic methylation, histone occupancy, and gene expression tested (*blue*), and proportions of the tested sites that passed significance *p* < 0.05 (*red*). **f** The percentage of allelic differentially modified histone sites as identified by GSCI that have an ASM ratio at the SNP-associated CpG in the top 1% versus the percentage of histone sites not having an allelic effect detected by GSCI but having an ASM ratio at the SNP-associated CpG in the top 1%

We also found that the largest proportion of significant interactions occurred when the high chromatin modification rate via H3K27ac, H3K4me1, and H3K4me3 tracked with high gene expression and were found on the low methylation allele (Fig. 6a). This is consistent with the roles of these chromatin modifications as activating and enhancer marks and the methylation indicating repression of transcription. Conversely, we observed for the more repressive H3K27me3, H3K36me3, and H3K9me3 marks that the high histone occupancy chromosome was the same as the high methylation chromosome and was the allele having lower gene expression. We again observed a strong effect for the activating and enhancer marks in the WGBS data, while the more repressive marks did not show a strong, consistent pattern. Using the MCC-Seq data we then compared the distribution amongst the different combinations of direction of effect, considering all levels of differential methylation, expression, and histone, and the cases where the differential effects were significant (*p* < 0.05) for methylation, expression, and histone simultaneously (Fig. 6b–e). Here we found that while we tested roughly the same proportion of allelic sites for all four possible allelic combinations, the enrichment for the canonical direction of effect is particularly strong. For activating H3K27ac (Fig. 6b) and H3K4me1 (Fig. 6c), we observed high methylation and low histone mark corresponding to low expression as the strongest signal. For repressive H3K36me3 (Fig. 6d) and H3K27me3 (Fig. 6e), we see high methylation and high histone corresponding to low expression as the strongest signal instead. We also used the genotype-independent signal correlation and imbalance (G-SCI) ASH test, and found that SNPs linked to ASH are enriched for association to allelic differential methylation, further strengthening the links between these allelic effects (Fig. 6f).

### Positional allelic methylation in enhancer regions

**Fig. 7:**
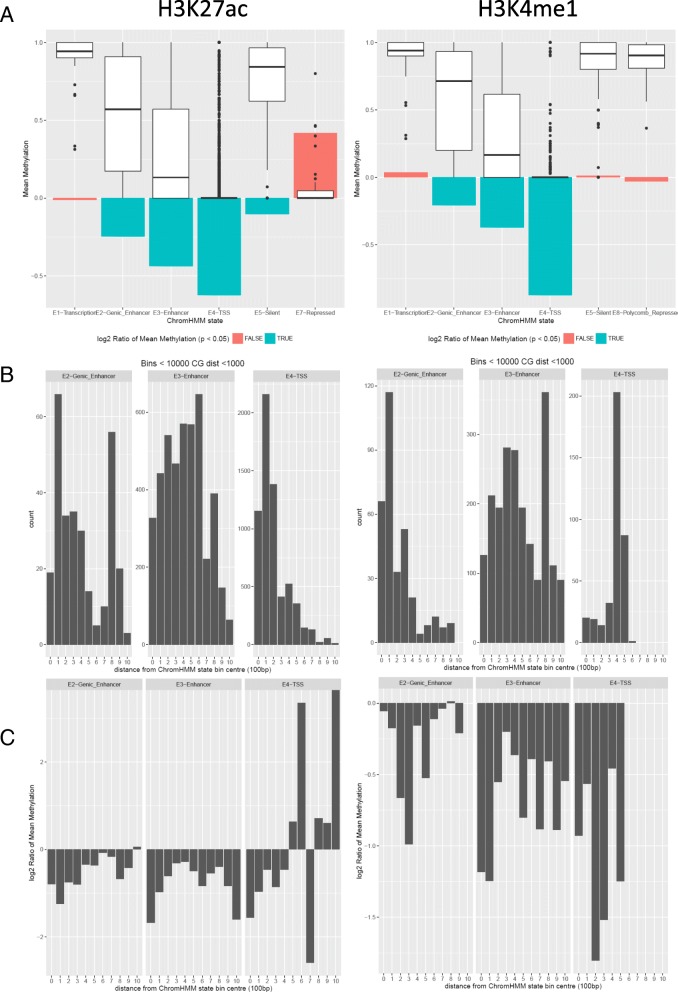
**a** The distribution of the mean methylation of CpGs by ChromHMM state, for CpGs in ChromHMM states where we see high correlation between gene expression and H3K27ac (*left*) or H3K4me1 (*right*) histone peaks. Bar graphs show the log2 mean allelic ratio of the methylation on the high gene expression allele versus the low gene expression allele (*green*; indicating *p* < 0.05) **b** Positional distribution of CpGs by distance from the center of the ChromHMM state, for all the ChromHMM states having at least 100 CpGs **c** Summary of log2 mean allelic methylation ratio of CpGs at each distance from the center of the ChromHMM state bin, for the high gene expression allele versus low gene expression allele

Finally, we focused on the enhancer signals in ChromHMM states—specifically, regions where we detect correlation between gene expression and H3K27ac or H3K4me1 peaks. We note that CpGs have overall high mean methylation (>75%) in transcription and silent states, low mean methylation (<25%) in the TSS state, and intermediate methylation in genic enhancer and enhancer states (Fig. 7a; Additional file 7). We also observed high methylation for the Polycomb repressed state, detected only in H3K4me1, and low methylation in the repressed state, only seen in H3K27ac, highlighting the different locations of these chromatin modifications. When we look at the methylation on the allele with high gene expression versus methylation on the low gene expression allele, we see a strong, consistent negative relationship between gene expression and methylation for genic enhancer, enhancer, and especially TSS states, which is consistent with the role of methylation marking the regulation of gene expression.

We note that many of the CpGs are located near the enhancer state for both H3K27ac and H3K4me1 (Fig. 7b), and both marks show most of the CpGs involved at around 300–500 bp from the center of the ChromHMM state bin. In the case of H3K27ac, there is also significant presence in the TSS state, concentrated in the first 300 bp from the center of the ChromHMM state bin. The CpGs in the genic enhancer state also showed a concentration slightly away from the center of the bin (100–400 bp) as well as further away from the center (800–900 bp). The majority of the H3K4me1 CpGs fall in the enhancer state, with some additional sites in genic enhancer and TSS states. Interestingly, we see the majority of the TSS state CpGs falling 400–500 bp away from the center, rather than near the center of the bin as with H3K27ac, indicating that regulatory element “edges” may be most informative to monitor for function. Finally, the genic enhancer CpGs are concentrated slightly away from the center of the bin (100–400 bp) as in H3K27ac, but without a second concentration further away from the center. These results show that not only can aggregated analyses detect enrichment of CpGs near functional genomic elements by combining with chromatin modification data, but that we can see distinctive, function-dependant positional patterns.

When looking at the positional effect of the GIT methylation (Fig. 7c), we see for H3K4me1 an overall negative ratio between the high and low expression allele, except for a loss of allelic methylation located 600–900 bp away from the genic enhancer state centers and 200–400 bp from the enhancer center. For H3K27ac, we again see the canonical negative methylation ratio between high and low gene expression alleles, except very distinctly at 500–700 bp from the center of the TSS states where we see a very strong spike in positive ratios.

## Discussion

We report a comprehensive integrative analysis from multiple large-scale human epigenomic datasets, across methylation, transcription, and chromatin modifications. For the first time we used tests that integrate allelic events across populations linked either to the reference allele or purely to recurrent imbalanced methylation states of chromosomes observable only in population NGS data. We have not only generated the largest catalogue of methylation changes in haploid human genomes, but by combining these three levels of genomic data with allelic profiling, we reveal novel relationships between genetic and non-genetic allelic variation. Non-genetic effects are seen in imprinted regions of the human genome but are also strikingly enriched in classes of developmental transcription factors. The non-genetic variation appears to have less involvement in active allelic chromatin states (28% of functional AS CpG–gene correlations). Our CpG allelic variation catalogue can be utilized to intersect with other population variation datasets to interrogate potential epigenetically variable (e.g., partial imprinting) regions in the genome. However, our allelic methylation observations are based on terminally differentiated cells and tissues, whereas these effects may be rooted in epigenetic memory of allelic exclusion events not active in the studied cells. The ~350,000 allelic events not driven by detectable genetic or known imprinted events call for further studies in developmental cell lineages to further clarify their potential functional roles.

We show that allelic CpG methylation and gene expression analysis allows for more sensitive detection of functional epigenetic effects and has the ability to reveal correlations not seen in non-allelic methylation to gene expression analyses. As each allele can contribute a separate methylation/expression data pair, allelic analysis has the advantage of potentially doubling the number of data points usable for the correlation analysis. As well, it is particularly interesting for disentangling relationships at hemi-methylated sites, where intermediate levels of gene expression and methylation may confound non-allelic methylation and expression analysis, but resolving the alleles separately shows different methylation rates and gene expression rates. While non-allelic analysis of MCC-seq data still results in more significant correlated sites than non-allelic analysis, most of the additional non-allelic correlations are far weaker—the bulk of the non-allelic correlations have |R| < 0.4, whereas over half the allelic correlations have |R| > 0.4. This trend is replicated when we look in the WGBS dataset, where there are distinctly far fewer significantly correlated sites and the correlations are higher when passing significance due to the smaller numbers of samples involved.

However, allelic analysis comes at the price of a reduced read depth for the individual alleles, thereby increasing the error rates in the allelic methylation and expression rate estimation. We observe that the allelic analysis of the lower coverage WGBS data shows fewer significant highly correlated sites, highlighting the impact of lower read depth coverage when profiling all CpGs genome-wide. Conversely, when examining the MCC-Seq data—where the read coverage is focused on a much smaller subset of the genome and we have a larger number of samples—we are able to identify a far greater number of significant, highly correlated sites. Developing deeper and larger WGBS datasets will be useful to further investigate some of our observations, such as primary concentration of epigenetic variation outside canonical regulatory elements. Further meta-analyses, incorporating additional eQTL/ASE, mQTL/ASM and hQTL/AS-ChIP sequencing datasets, would allow us to independently confirm the same relationships in similar cells, determine whether the observed relationships are maintained in more distantly related cell types, as well as further differentiate cell type-specific patterns. Corresponding sequencing datasets in other species would allow us to investigate the evolutionary conservation of these multi-level effects.

The large number of relationships detected uniquely by allelic or non-allelic analysis alone indicates that these analyses can identify fundamentally different relationships between the epigenome and the transcriptome. Non-allelic correlation between methylation and gene expression focuses on global trends of methylation expression between cells affecting global gene expression levels. Allelic correlation, however, normalizes the gene expression across both alleles in a cell, and so the trend is no longer across the total gene expression between cells, but rather a normalized allelic imbalance of expression, as affected by allelic methylation. We see that while allelic functional methylation analysis is highly sensitive and reveals many non-canonical relationships outside regulatory elements. On the other hand, the subset of variation in enhancers/promoters coinciding with allelic active histone deposition results in a very coherent picture that shows the expected relationships between the epigenetic layers of control and functional gene expression. These strong, consistent patterns indicate that combined allelic analysis of multiple epigenetic layers allows us to track interactions with direct functional effects.

We also see the canonical relationship between methylation and gene expression in an allelic manner, with the high gene expression allele being coincident with the low methylation allele. However, we see a positional loss of this canonical relationship (in H3K4me1) or even reversed to a positive relationship (in H3K27ac), a short distance from the center of the enhancer feature (Fig. 7c). The loss of allelic methylation may delineate the boundary of the enhancer region and the adjacent “marginal” CpGs can actually serve as sensors of enhancer element function, showing strongest correlation with expression states. Interestingly, it appears that the promoters of genes with expression coupled to H3K27ac deposition have distinctly different positional methylation architecture, with a larger density of CpGs clustered by the center of the TSS region, whereas for H3K4me1 the CpGs are grouped (Fig. 7b).

## Conclusions

We combine allelic analysis across multiple epigenomic and transcriptomic layers, revealing interactions between the varied layers of the effects. Models that can simultaneously use the allelic and non-allelic data could allow us to supplement the non-allelic analysis with allelic information when it is available, rather than analyzing this information in a completely disjoint manner, and without losing the relationships that allelic or non-allelic analyses alone can discover. As it has been shown that correlations link many layers of functional signals in an allele-specific manner in more limited contexts (e.g., GM12878 cells [35]), further analyses could go beyond coordinating three layers of effects and consider multiple histone marks and transcription factor binding simultaneously with gene expression and methylation in an allelic manner in a population context.

At present, we are using non-allelically resolved histone mark datasets to train the ChromHMM model, and we show different behavior of the epigenome and transcriptome in different states. We could more tightly integrate allelic data into the genomic state analysis by adapting the allelic information and incorporate allele-specific epigenomic information in the ChromHMM inputs so that the states themselves take into account allelic information, while integrating across multiple epigenome and transcriptome layers, and see how this affects the model of the genome that is generated. As well, our case study demonstrates how the allelic relationships between methylation and histone marks are altered in the different forms of the disease, further emphasizing the future utility of allelic resolution in disentangling the functional outcome. Finally, more sequencing in distinct tissues and cell types would allow us to confirm the preliminary cell-specific methylation results we observe as well as allow us to investigate the interaction of cell-specific effects across the epigenetic layers.

In summary, we have generated the largest combined human allele-specific methylation, chromatin modification, and transcription dataset to date (Additional files 2, 3, 4, 6, and 7). We show allelic methylation enables discovery of novel links to transcription, whereas total methylation shows weaker correlations in layers of the epigenome. We further demonstrate that harnessing the power provided by allelic resolution across methylation, transcription, and chromatin modification is key to interpreting population variation in our epigenomes and its alterations in disease.

## Methods

### Sample collections

We recruited 208 donors from the Cambridge NIHR BioResource as a representative sample of healthy individuals from the general UK population. Whole blood was used to purify “classic” naive CD4+ T cells (CD4 + CD45RA+, average purity 93%) using a multi-step purification strategy. Purified cell aliquots were pre-processed, stored, and transported to processing institutes for sequencing as previously described [34].

We obtained 114 visceral adipose tissue (VAT) samples from the Quebec Heart and Lung Institute’s (Quebec City, Quebec, Canada) Quebec Tissue Bank collection of 1906 severely obese men (N = 597) and women (N = 1309) that underwent biliopancreatic diversion with duodenal switch [36] between June 2000 and July 2012. The VAT samples were obtained as previously described [37] and processed for methylation sequencing as reported in Allum et al. [21] and below.

We obtained 599 whole blood samples from 358 individuals in the framework of the *EGEA* (https://egeanet.vjf.inserm.fr/), a French longitudinal cohort study based on an initial group of asthma cases and their first-degree relatives and controls (first survey EGEA1). The protocol and descriptive characteristics have been described previously [38–42].

Blood samples from the Uppsala Bioresource, Uppsala, Sweden were drawn from 28 normal healthy Swedish individuals and purified to extract T cells (CD14− CD4+) and monocytes (CD14+). Nine skeletal muscle human samples were also obtained. Sequencing data are available through the McGill Epigenomics Mapping Portal (http://epigenomesportal.ca).

These samples and the sequencing datasets derived from them are described globally in Fig. 1, with further detail specifying the cell types and mean read depth available for each dataset in Additional file 5: Figure S2 and Additional file 8: Table S1. At each step, we performed tests on each dataset (all samples of a particular cell type and methylation sequencing methodology combination, matched with RNA-Seq and ChIP-Seq if needed) and report the combined results of these tests.

### Methylation sequencing

Targeted bisulfite sequencing (MCC-Seq) and whole genome bisulfite sequencing was performed as previously described [21]. A whole-genome sequencing library is prepared and bisulfite converted, amplified and a capture enriching for targeted bisulfite-converted DNA fragments is carried out. The captured DNA is further amplified and sequenced. More specifically, whole-genome sequencing libraries were generated from 700 to 1000 ng of genomic DNA spiked with 0.1% (w/w) unmethylated λ DNA (Promega) previously fragmented to 300–400 bp peak sizes using the Covaris focused-ultrasonicator E210. Fragment size was controlled on a Bioanalyzer DNA 1000 Chip (Agilent) and the KAPA High Throughput Library Preparation Kit (KAPA Biosystems) was applied. End repair of the generated dsDNA with 3′ or 5′ overhangs, adenylation of 3′ ends, adaptor ligation, and clean-up steps were carried out as per KAPA Biosystems’ recommendations. The cleaned-up ligation product was then analyzed on a Bioanalyzer High Sensitivity DNA Chip (Agilent) and quantified by PicoGreen (Life Technologies). Samples were then bisulfite converted using the Epitect Fast DNA Bisulfite Kit (Qiagen) according to the manufacturer’s protocol. Bisulfite-converted DNA was quantified using OliGreen (Life Technologies) and, based on quantity, amplified by 9–12 cycles of PCR using the Kapa Hifi Uracil + DNA polymerase (KAPA Biosystems) according to the manufacturer’s protocol. The amplified libraries were purified using Ampure Beads, validated on Bioanalyzer High Sensitivity DNA Chips, and quantified by PicoGreen.

For targeted bisulfite sequencing, we used the MCC-Seq protocol developed and optimized in collaboration with Roche NimbleGen R&D. SeqCap Epi Enrichment System protocol (Roche NimbleGen) was carried out for the capture. The hybridization procedure of the amplified bisulfite-converted library was performed as described by the manufacturer using 1 μg of total input of library, which was evenly divided by the libraries to be multiplexed, and incubated at 47 °C for 72 h. Washing and recovering of the captured library, as well as PCR amplification and final purification, were carried out as recommended by the manufacturer. The quality, concentration, and size distribution of the captured library were determined by Bioanalyzer High Sensitivity DNA Chips.

Sequencing of both the MCC-Seq libraries and the WGBS libraries was performed on the Illumina HiSeq2000/2500 system using 100-bp paired-end sequencing.

### WGBS and MCC-Seq data processing

In-house generated methylome libraries were aligned using BWA 0.6.1 [43] after converting all the reads in bisulfite mode to the human hg19/GRCh37 genome reference. Both reads in a pair were trimmed of any low-quality sequence at their 3′ ends (with Phred scale score ≥30). Post-process read mappings were made as previously described [44], including clipping 3′ ends of overlapping read pairs in both forward and reverse strand mappings, filtering duplicate, low-mapping quality reads, read pairs not mapped at the expected distance based on the library insert size, as well as reads with more than 2% mismatches. Methylation calls of individual CpGs were extracted using Samtools in mpileup mode. CpGs overlapping SNPs from dbSNPs (137) and CpGs located within ENCODE DAC blacklisted regions or Duke excluded regions [35] were discarded. CpGs with the number of total reads less than 5× were also discarded.

### Genotyping and phased genome

The T-cell, mono cell (in Temporal Change project), and muscle tissue samples were genotyped using the Illumina HumanOmni2.5-8 (Omni2.5 M) or HumanOmni5-4 (Omni5M) BeadChip according to protocols recommended by Illumina. Genotypes of BluePrint samples were obtained from whole genome sequencing [34]. Genotypes of samples from other tissues which were not genotyped using BeadChip were inferred directly from the WGBS data using BisSNP [45]. Rare variants and singletons were confirmed using targeted sequencing data of coding and non-coding regulatory regions.

We used 1000 Genomes project data as a reference set (release 1000G Phase I v3, updated 26 Aug 2012) for the imputation of genotypes (either genotyped from Illumina BeadChip or inferred from BisSNP). Untyped/un-inferred markers were inferred using algorithms implemented in IMPUTE2 [46].

mQTLs were computed using matrixQTL using default parameters, considering only *cis*-effects within 250 kb and minor allele frequency of 0.05. *P* values were corrected by false discovery rate (FDR) [47].

### RNA-Seq

RNA-Seq was performed as described previously. RNA was isolated using the miRNeasy Mini Kit (Qiagen) according to the manufacturer’s protocol. We used as input 500 ng RNA (RNA integrity number >7) for library preparations using the Illumina TruSeq Stranded Total RNA Sample preparation kit according to the manufacturer’s protocol. Final libraries were quality controlled on a Bioanalyzer and underwent 100-bp paired-end sequencing on the Illumina HiSeq2000 system. Generated raw reads were filtered for quality (phred33 ≥ 30) and length (n ≥32), and adapter sequences were removed using Trimmomatic v.0.32 [48]. Reads passing filters were then aligned to the human reference (hg19) using TopHat v.2.0.10 [49] and bowtie v.2.1.0 [50]. UCSC gene counts for non-allelic analysis were obtained using htseq-count v.0.6.1 [51].

### ChIP-Seq

Sonication of nuclei was performed on a BioRuptor UCD-300 for 90 cycles, 10 s on, 20 s off, centrifuged every 15 cycles, chilled in a 4 °C water cooler. Samples were checked for sonication efficiency using the criteria of 150–500-bp by gel electrophoresis. The ChIP reaction was performed on a Diagenode SX-8G IP-Star Compact using Diagenode automated Ideal Kit reagents (C01010011). Protein A beads (25 μL) were washed and then incubated with 3–6 μg of antibody and two to four million cells of sonicated cell lysate combined with protease inhibitors for 10 h, followed by a 20-minute wash cycle with provided wash buffers. Reverse cross-linking took place on a heat block at 65 °C for 4 h. ChIP samples were then treated with 2 μl RNAse cocktail at 65 °C for 30 minutes followed by 2 μL Proteinase K at 65 °C for 30 minutes. Samples were then purified with a Qiagen MiniElute PCR purification kit as per the manufacturers’ protocol. Library preparation was carried out using Kapa HTP Illumina library preparation reagents. Briefly, 25 μl of ChIP sample was incubated with 20 μl end repair mix at 20 °C for 30 minutes followed by Ampure XP bead purification. A tailing, bead-bound sample was incubated with 50 μL buffer enzyme mix at 30 °C for 30 minutes, followed by PEG/NaCl purification. Adapter ligation, further Ampure purification, and library preparation were completed by 14 cycles of PCR amplification. Size selection was performed using a Sage Pippin prep system and set to collect 200–400-bp fragments, targeting a 300-bp peak fragment size and final libraries were purified with Qiagen GeneRead Size Selection kit.

ChIP libraries were sequenced at McGill using Illumina HiSeq 2000 with 100-bp single-ended reads. Generated raw reads were filtered for quality (phred33 ≥ 30) and length (n ≥ 32), and adapter sequences were removed using Trimmomatic v.0.22 [52]. Reads passing filters were then aligned to the human reference (hg19) using BWA v.0.6.1. Peak calls were obtained using MACS2 v.2.0.10.07132012 [4].

### Allele-specific methylation pipelines

Imputed and phased genotypes were used to create two allele-specific copies of the reference genome. Reads were then mapped to these two reference genomes using the sample pipeline as described above [44], except that no mismatches were allowed during the alignment step in order to ensure that reads coming from a specific allele will map to the appropriate reference. An in-house software takes the genotypes and their positions and scans the alignment files to obtain the methylation states of the CpGs surrounding the alleles. The width of the area scanned spans 500 bp upstream and downstream of a heterozygous SNP. For the cases where imputed phased genomes are not available, we considered the methylation status of CpGs from paired-end reads containing the same heterozygous SNP (as inferred by BisSNP). As a strand-specific WGBS protocol, reads mapped from both strands were counted. When there is a “C/T” heterozygous SNP neighboring a CpG, only reads from the reverse strand were considered while for a “G/A” heterozygous SNP, only reads from the forward strand were considered. In order to appropriately estimate methylation levels, a bare minimum number of five reads per allele (from both strands) is required. We then summed the reads across all individuals based on alleles’ genotyping to detected genetic ASM. After obtaining the methylation states of individual CpGs per allele, the differential methylation between the two alleles at CpGs were then determined using a two-sided Fisher’s exact test. CpGs with *p* < 0.05 were considered nominally significant.

For each cell type, we performed a test of methylation imbalance (GIT) by merging allele-specific methylation reads across samples. At a CpG, we categorized one allele per sample as the high methylation allele and the other as the low methylation allele. We then considered the total methylated and total unmethylated reads for the high methylation alleles, and similarly for the low methylation alleles. In this way, we obtained sample-merged read counts of individual CpGs per allele. Differential methylation between high and low methylation alleles at CpGs was then determined using a two-sided Fisher’s exact test. CpGs showing *p* < 0.05 were considered nominally significant.

To determine significant ASM and mQTL sites, we applied FDR [47] at *q* < 0.1. For GIT, we performed a one-sided Fisher’s exact test, corrected by the total probability of all possible contingency tables where methylation on the high methylation allele is greater than the low methylation allele. We then applied a more stringent FDR cutoff of *q* < 0.01 to identify the significant GIT sites. Putative epigenetically regulated CpGs are significant imbalanced CpGs (corrected *q* < 0.01) having no significant genetic methylation (ASM and mQTL *q* ≥ 0.1).

### Allele-specific chromatin modifications

Reads from ChIP-seq data were trimmed for quality (phred33 ≥ 30) and length (n ≥ 32) using Trimmomatic v.0.22 [52]. The filtered reads were aligned to the hg19 reference genome using BWA v.0.61. We then binned genomic regions with 100 bp-window to get the aligned read counts and arbitrarily chose top 300 K of 100-bp bins as the candidate peaks for different types of histone markers, including H3K27ac, H3K27me3, H3K36me3, H3K4me1, H3K4me3, and H3K9me3. When calculating the allele-specific marker regions, for each heterozygous SNP within the region we counted the number of reads of different origin that overlapped with the SNPs. The allele bias was then tested using a binomial test against the null hypothesis that the ratio between these two alleles is equal. SNPs with *p* < 0.05 were considered allele-specific. The allelic ratio for the high methylation allele was computed as 0.5-fraction of ChIPSeq reads on the high methylation allele compared to the total (high and low allele) reads, and similarly for the low methylation allele using the fraction of ChIPSeq reads on the low methylation allele.

### Gene expression versus RNA expression correlations

Non-allelic methylation was measured as the percentage of methylated CpG reads compared to the total methylated and unmethylated reads overlapping the CpG site. Allelic methylation considers only reads with a heterozygous SNP that could be resolved to one of the two chromosomes:

$$ Methylatio{n}_{alleleA}=\frac{unmethylatedRead{s}_{alleleA}}{unmethylatedRead{s}_{alleleA}+ methylatedRead{s}_{alleleA}} $$

Non-allelic gene expression was measured as the library size and quantile normalized, asinh transformed read counts of aligned RNA reads:

$$ N o n A l l e l i c E x p r e s s i o n=\mathrm{asinh}\left(\mathrm{quantileNormalised}\left(\frac{GeneReads}{TotalReads}\right)\right) $$

Allelic gene expression only considered reads resolved to one of the two chromosomes. Gene expression for an allele A was the number of reads aligning to allele A divided by the total of reads aligning to allele A and reads aligning to allele B.

$$ AllelicExpressio{n}_{alleleA}=\frac{read{s}_{alleleA}}{read{s}_{alleleA}+ read{s}_{alleleB}} $$

Pearson correlation was used to evaluate relationships between CpGs and genes with TSS within 50 kb of the CpGs, for cases where at least three samples had matching gene expression and methylation data.

### ChromHMM genomic states

An eight-state ChromHMM [25] model trained using default parameters on a panel of 352 T-cell, monocyte, and muscle histone (H3K27ac, H3K27me3, H3K36me3, H3K4me1, H3K4me3, and H3K9me3) ChIP-Seq datasets was used to assign states to each CpG position. State identities were assigned based on the ChromHMM report (Additional file 9).

### G-SCI test

We also performed allelic regulatory QTL analysis on multiple histone marks using a recent published method called the genotype-independent signal correlation and imbalance (G-SCI) test [53]. It was originally designed for detecting histone acetylation QTLs (haQTLs) from deep, long-read ChIP-seq data without requiring genotyping or whole genome sequencing. It first called variants with base calling information directly from the ChIP-seq sequence reads in peaks and then correlated these variants with chromatin states to prioritize the genetic variants. The G-SCI test itself only required variant base calling information and the peak height score information; thus, it can also apply to detect the association between any histone marker peak regions and concerned variants within peak regions. We applied the G-SCI test to all available histone mark ChIP-Seq data with SNPs detected from imputed genotypes of each corresponding individual. Peak heights of each region were normalized by quantile-quantile normalization and were finally log-transformed. We also filtered out SNPs if the total number of non-reference reads across all ChIP-Seq data was less than five or none of the ChIP-seq datasets had three or more non-reference reads. After obtaining the *p* value of each G-SCI test, the Benjamini and Hochberg (BH) approach for FDR [54] was used to correct for multiple testing and the adjusted *p* value of 0.01 was chosen as the significant cutoff.

### ASM versus ASH

We mapped heterozygous SNP-based histone marker read counts from two alleles to CpGs by matching with the same SNPs. For GIT, when the imputed phased genome was available, then ASH counts of SNPs 500 bp away from the corresponding CpGs were added up. Otherwise, when an imputed phased genome was not available and for ASM, only the ASH counts of the same SNPs were considered.

### Genome feature association

Based on human genome hg19, annotation tables of genomic features were downloaded from the UCSC Genome Browser [55] on 10 September 2013. Overlapping between any two regions were calculated using bedtools [56].

### Positional allelic methylation in enhancer regions

Focusing on the deposition of the enhancer histone marks H3K27ac and H3K4me1, we linked to allelic gene expression, histone deposition, and methylation back to the genomic states and looked at the positional pattern of allelic methylation. We identified ChromHMM state regions where we observed a correlation (|R| > 0.4, *p* < 0.05) between gene expression and the histone mark deposition in nTC cells (100-bp windows from the center of H3K27ac peaks, 500-bp windows for H3K4me1 peaks) overlapping the state region, considering ChromHMM state regions up to 10 kb in size. For each of these putative enhancer regions, we then phased the allelic methylation for all CpGs in the region based on the high gene expression and the low gene expression allele—for genes with multiple transcripts, we selected the transcript isoform with the highest number of exonic reads, breaking ties with the largest overall gene reads. CpGs were grouped based on their absolute distance from the center of the bin in 100-bp increments, calculating overall methylation on each allele for each bin separately.

### Disease GWAS enrichment of ASM event

We used GWAS variants from eight diseases (celiac disease [27], Crohn’s disease [28], inflammatory bowel disease [28], ulcerative colitis [28], multiple sclerosis [29], rheumatoid arthritis [30], type 1 diabetes [31], and type 2 diabetes [32]) for which we retrieved publicly available genome-wide summary statistics [34]. We tested genome-wide enrichment for independent variants (LD r^2^ < 0.1) nominally associated with disease (*p* value ≤10^−5^) among significant ASM-SNPs (*q* < 0.1) or with a SNP in high LD (r^2^ > 0.8). The background was defined as all independent GWAS SNPs tested overlapping any SNPs’ ASM that was tested. The significance of the enrichment was assessed using Fisher’s exact test. LD information was calculated for each SNP ±250 kb using phased data from whole genome sequencing of the whole blood samples.

## Additional files

Additional file 1: Description of MCC-Seq capture panel. This file contains: (1) summary of CpGs and genomic regions targeted by the MCC-Seq capture panel design (.xlsx Excel spreadsheet format), and (2) a list of the targeted regions (.bed text file). (ZIP 3465 kb)
Additional file 2: Summary of AS and NAS methylation tests on the 2.2 million CpGs tested across any of the datasets. This file contains a file of tab-separated values (.txt text file) with the following columns: (1) CpG chromosome, (2) CpG location, (3) corrected GIT *p* value, (4) corrected mQTL *p* value, (5) corrected ASM *p* value, (6) ChromHMM state. (ZIP 19804 kb)
Additional file 3: High-confidence non-genetic CpG regions. This file contains a list of genomic regions (.bed text file) containing at least 15 consecutive CpGs and where all CpGs did not show significant genetic methylation (ASM and mQTL *q* ≥ 0.1) but showed significant imbalanced allelic methylation (GIT *q* < 1 × 10^−5^), and where the median imbalanced allelic methylation was highly significant (log_e_(*q*) < −10). (ZIP 8 kb)
Additional file 4: GREAT analysis results of the high-confidence non-genetic CpG regions. This file lists over-representation analysis results from GREAT for GO biological process, Interpro, and HGNC Gene Families. For the GO biological process analysis, child terms of the GO term “developmental process” are highlighted in *green*, and child terms of the GO term “metabolic process” are highlighted in *yellow*. For Interpro and HGNC gene families, homeobox/homeodomain genes are highlighted in *green* and cadherin/adhesion genes are highlighted in *yellow*. (XLSX 17 kb)
Additional file 5: Figure S1. Validation using 450 k array. **Figure S2.** Detailed description of samples per cell type. (PDF 440 kb)
Additional file 6: AS and NAS CpG methylation to RNA expression correlations. This mini-website describes the file format and links to individual correlation results for each cell type (.txt tab-separated text file). (ZIP 18638 kb)
Additional file 7: Allelic methylation in enhancer peaks. In this file, we report the ChromHMM bin and the allele-sepcific CpG methylation and RNA read coverage at CpGs within H3K27ac and H3K4me1 ChIP-Seq peaks. (XLSX 1899 kb)
Additional file 8: Table S1. This file details for each cell type mean fold coverage (for CpG methylation sequencing) and total aligned reads (for RNA-Seq and ChIP-Seq). (XLSX 10 kb)
Additional file 9: ChromHMM state report. This file is the report generated after machine learning of the eight-state ChromHMM model. (PDF 261 kb)
Additional file 10: Source code. This file contains the code for generating the ASM *p* values: Get_CpG.SNP.pairs.mergedSamples.Profile.PhasedAlleles.pl (.pl perl code script), Get_ASMsite_FisherTesting.PhasedAlleles.MergedReads.r (.R R statistics script); the code for generating the GIT *p* values: Get_CpG.SNP.pairs.mergedSamples.Profile.FlippedHL.pl (.pl perl code script), Get_ASMsite_FisherTesting.FlippedHL.MergedReads.r (.R R statistics script); and the code for the GIT *q* value statistic correction: GIT-qvalue.correction.R (.R R statistics code script). (ZIP 8 kb)
