# Correction to: Functional variation in allelic methylomes underscores a strong genetic contribution and reveals novel epigenetic alterations in the human epigenome

**DOI:** 10.1186/s13059-019-1702-7

**Published:** 2019-05-07

**Authors:** Warren A. Cheung, Xiaojian Shao, Andréanne Morin, Valérie Siroux, Tony Kwan, Bing Ge, Dylan Aïssi, Lu Chen, Louella Vasquez, Fiona Allum, Frédéric Guénard, Emmanuelle Bouzigon, Marie-Michelle Simon, Elodie Boulier, Adriana Redensek, Stephen Watt, Avik Datta, Laura Clarke, Paul Flicek, Daniel Mead, Dirk S. Paul, Stephan Beck, Guillaume Bourque, Mark Lathrop, André Tchernof, Marie-Claude Vohl, Florence Demenais, Isabelle Pin, Kate Downes, Hendrick G. Stunnenberg, Nicole Soranzo, Tomi Pastinen, Elin Grundberg

**Affiliations:** 1 Department of Human Genetics, McGill University, Montreal, Quebec Canada; 2 McGill University and Genome Quebec Innovation Centre, Montreal, Quebec Canada; 3 Team of Environmental Epidemiology Applied to Reproduction and Respiratory Health, Inserm U1209, CNRS, University Grenoble Alpes, Institute for Advanced Biosciences, Grenoble, France; 4 Department of Human Genetics, The Wellcome Trust Sanger Institute, Wellcome Trust Genome Campus, Hinxton, Cambridge, CB10 1HH UK; 5 Department of Haematology, University of Cambridge, Cambridge Biomedical Campus, Long Road, Cambridge, CB2 0PT UK; 6 Institute of Nutrition and Functional Foods (INAF), Laval University, Québec, QC G1V 0A6 Canada; 7 Genetic Variation and Human Diseases Unit, UMR-946, INSERM, Université Paris Diderot, Université Sorbonne Paris Cité, Paris, France; 8 European Molecular Biology Laboratory, European Bioinformatics Institute, Wellcome Genome Campus, Hinxton, Cambridge, CB10 1SD UK; 9 UCL Cancer Institute, University College London, 72 Huntley Street, London, WC1E 6BT UK; 10 Cardiovascular Epidemiology Unit, Department of Public Health and Primary Care, University of Cambridge, Strangeways Research Laboratory, Worts Causeway, Cambridge, CB1 8RN UK; 11 Québec Heart and Lung Institute, Laval University, Québec, QC G1V 4G5 Canada; 12 Pédiatrie, Centre Hospitalier Universitaire (CHU) Grenoble Alpes, Grenoble, France; 13 National Health Service (NHS) Blood and Transplant, Cambridge Biomedical Campus, Long Road, Cambridge, CB2 0PT UK; 14 Faculty of Science, Department of Molecular Biology, Radboud University, Nijmegen, 6525GA The Netherlands; 15 British Heart Foundation Centre of Excellence, Division of Cardiovascular Medicine, Addenbrooke’s Hospital, Hills Road, Cambridge, CB2 0QQ UK; 16 The National Institute for Health Research Blood and Transplant Unit (NIHR BTRU) in Donor Health and Genomics, University of Cambridge, Strangeways Research Laboratory, Wort’s Causeway, Cambridge, CB1 8RN UK

**Correction to: Genome Biol**

**https://doi.org/10.1186/s13059-017-1173-7**

Following publication of the original article [1], the authors reported an error in Additional file 1. The updated Additional file 1 is given below.

## Additional file

Additional file 1 Description of MCC-Seq capture panel. This file contains: (1) summary of CpGs and genomic regions targeted by the MCC-Seq capture panel design (.xlsx Excel spreadsheet format), and (2) a list of the targeted regions (.bed text file). (ZIP 4405 kb)
