# Supplementary material for: Functional variation in allelic methylomes underscores a strong genetic contribution and reveals novel epigenetic alterations in the human epigenome
Source: Genome Biol. 2017 Mar 10;18:50. doi: 10.1186/s13059-017-1173-7 (PMC5346261; doi:10.1186/s13059-017-1173-7)
Supplement: Additional file 5: Figure S1. — Validation using 450 k array. Figure S2. Detailed description of samples per cell type. (PDF 440 kb) [file 13059_2017_1173_MOESM5_ESM.pdf]

Figure S1. Validation using 450k array. (a) We show the density distribution of CpG methylation rates assayed using the 450k array across all sequenced CpG sites in the nTC samples, and across significant ASM sequenced CpG sites at various q-value thresholds. (b) Smoothed scatter plot of 450k array methylation vs. estimated methylation from the allele-specific methylation all the CpG sites. (c) Smoothed scatter plot of 450k vs. sequenced methylation at ASM CpG sites where ASM q < 0.1.

A

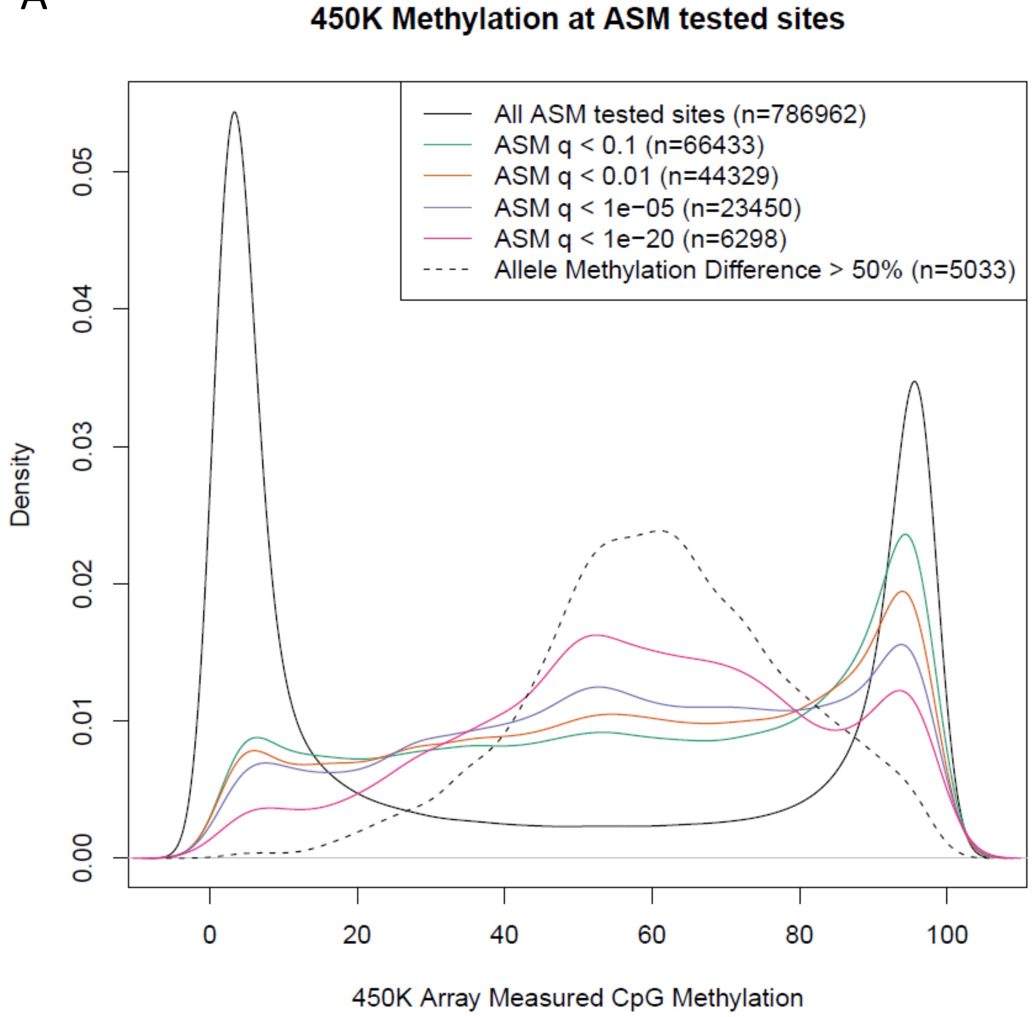

B

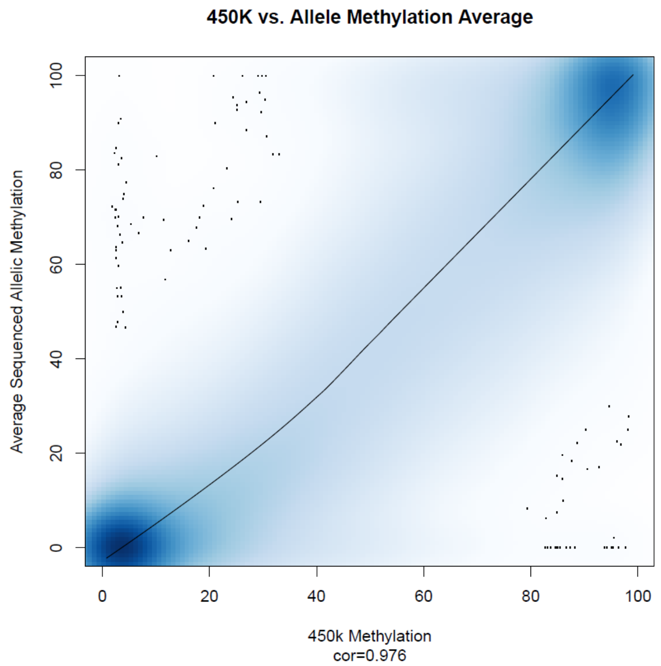

C

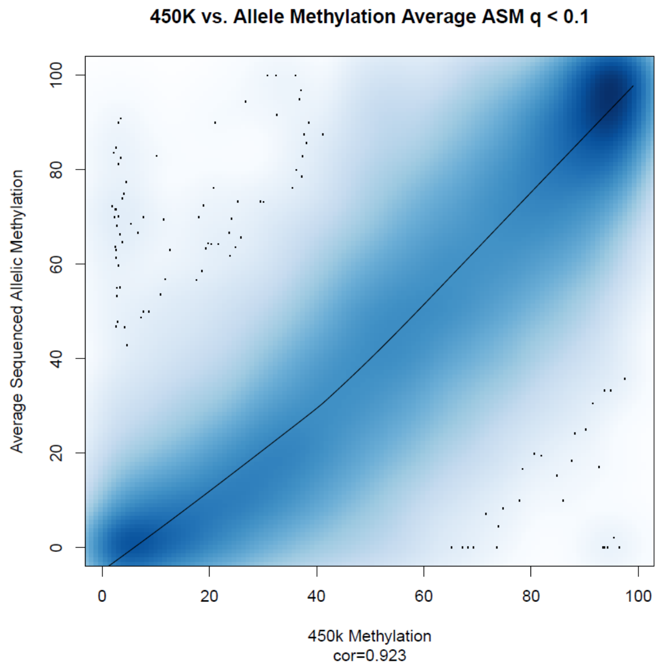

Figure S2. Detailed description of samples per cell types. Samples having multiple layer of epigenetics profiles were used in this project. Samples were all assessed for DNA methylation profile using either Whole-genome bisulfite sequencing (WGBS; in green) or methylC-capture sequencing (MCC-seq; in red). The number of samples used for analyses focusing only on DNA methylation alone, DNA methylation with Illumina 450K, DNA methylation with RNA-sequencing or DNA methylation with ChIP-Sequencing (using six different histone marks) and RNA-Sequencing are listed in this figure. We show the breakdown in each category by celltype – CD4+ T-Cells (TC), CD14+ Monocytes (Mono), Visceral Adipose Tissue (VAT) and Whole Blood (WB).

	**WGBS**	**MCC-seq**
A. DNA Methylation Alone	N=30 TC[1], N=6 Muscles, N=5 Mono[2]	N=148 VAT[3], N=599 WB[4], N=115 TC[1], N=7 Mono[2]
B . DNA Methylation with matched Illumina 450K		N=99 TC
C . DNA Methylation with matched RNA-Seq	N=30 TC[1], N=6 Muscles, N=5 Mono[2]	N=7 Mono, N=115 TC

D . DNA Methylation with matched ChIP-Seq & RNA-Seq

Mark	WGBS	MCC-seq
H3K4me1	N=24 TC, N=2 Mono, N=6 Muscles	N=2 Mono, N=94 TC
H3K27ac	N=24 TC, N=1 Mono, N=5 Muscles	N=2 Mono, N=107 TC
H3K4me3	N=16 TC, N=2 Mono, N=5 Muscles	N=2 Mono, N=1 TC
H3K9me3	N=14 TC, N= 1 Mono, N=5 Muscles	N=2 TC, N=2 Mono
H3K27me3	N=16 TC, N=2 Mono, N=6 Muscles	N=2 TC, N=2 Mono
H3K36me3	N=16 TC, N=2 Mono, N= 6 Muscles	N=2 TC, N=2 Mono
