# Supplementary material for: Functional variation in allelic methylomes underscores a strong genetic contribution and reveals novel epigenetic alterations in the human epigenome
Source: Genome Biol. 2017 Mar 10;18:50. doi: 10.1186/s13059-017-1173-7 (PMC5346261; doi:10.1186/s13059-017-1173-7)
Supplement: Additional file 6: — AS and NAS CpG methylation to RNA expression correlations. This mini-website describes the file format and links to individual correlation results for each cell type (.txt tab-separated text file). (ZIP 18638 kb) [file 13059_2017_1173_MOESM6_ESM.zip › Additional File 6 - ALL-PROJECT.short-stats.txt/index.html]

# Methylation/Gene Expression Correlations

Columns: CpG site, Position relative to TSS, Gene, Samples with
methylation, Samples with expression, Samples with matching
methylation and expression, NAS correlation, NAS p-value for correlation, mean
methylation, methylation variance, mean expression, expression
variance, AS correlation, AS p-value for correlation, ChromHMM bin
for CpG

- nTC WGBS- nTC MCC-Seq- Muscle WGBS- Monocyte WGBS- Monocyte MCC-Seq- T-cell WGBS- T-cell MCC-Seq
