# Supplementary material for: Functional variation in allelic methylomes underscores a strong genetic contribution and reveals novel epigenetic alterations in the human epigenome
Source: Genome Biol. 2017 Mar 10;18:50. doi: 10.1186/s13059-017-1173-7 (PMC5346261; doi:10.1186/s13059-017-1173-7)
Supplement: Additional file 9: — ChromHMM state report. This file is the report generated after machine learning of the eight-state ChromHMM model. (PDF 261 kb) [file 13059_2017_1173_MOESM9_ESM.pdf]

ChromHMM Report

Input Directory: output_tchange_muscle2
Output Directory: test_out_tchange_muscle2_8states
Number of States: 8
Assembly: hg19
Full ChromHMM command: LearnModel -p 4 -s 1234567890 output_tchange_muscle2 test_out_tchange_muscle2_8states 8 hg19

Model Parameters

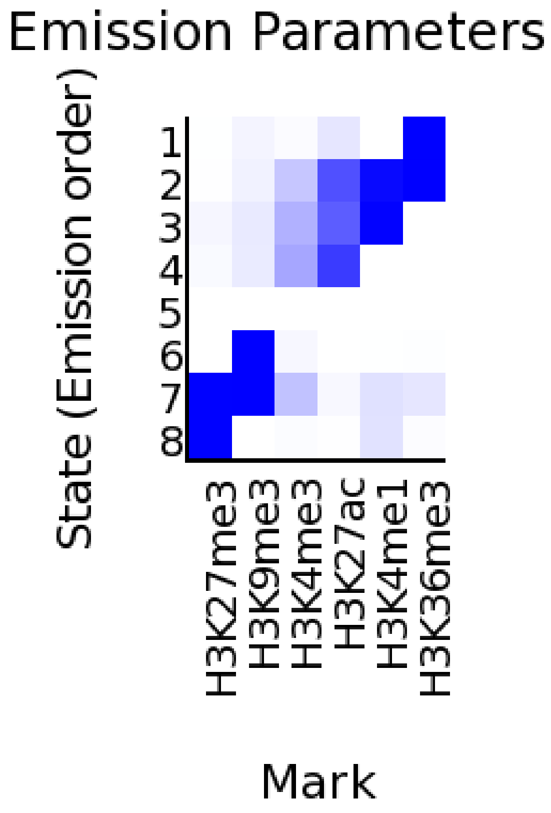

- Emission Parameter SVG File
- Emission Parameter Tab-Delimited Text File

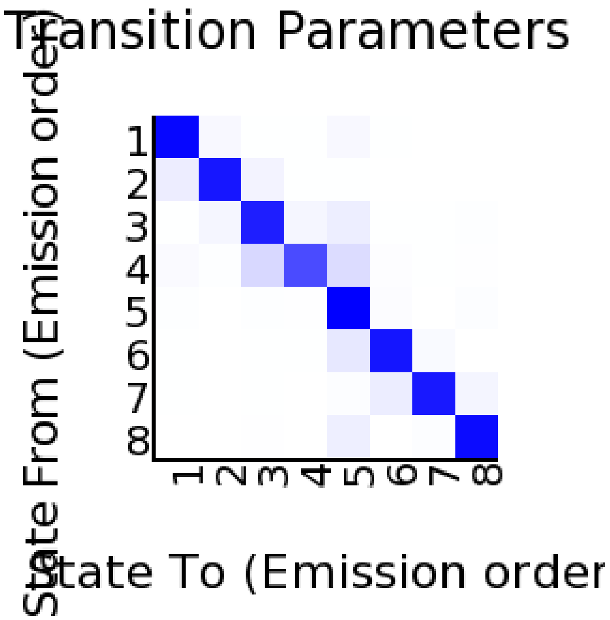

- Transition Parameter SVG File
- Transition Parameter Tab-Delimited Text File

- All Model Parameters Tab-Delimited Text File

Genome Segmentation Files

- Mono_8 Segmentation File (Four Column Bed File)
- Muscle_8 Segmentation File (Four Column Bed File)
- TC_8 Segmentation File (Four Column Bed File)

Custom Tracks for loading into the UCSC Genome Browser:

- Mono_8 Browser Custom Track Dense File
- Mono_8 Browser Custom Track Expanded File
- Muscle_8 Browser Custom Track Dense File
- Muscle_8 Browser Custom Track Expanded File
- TC_8 Browser Custom Track Dense File
- TC_8 Browser Custom Track Expanded File

State Enrichments

Mono_8 Enrichments

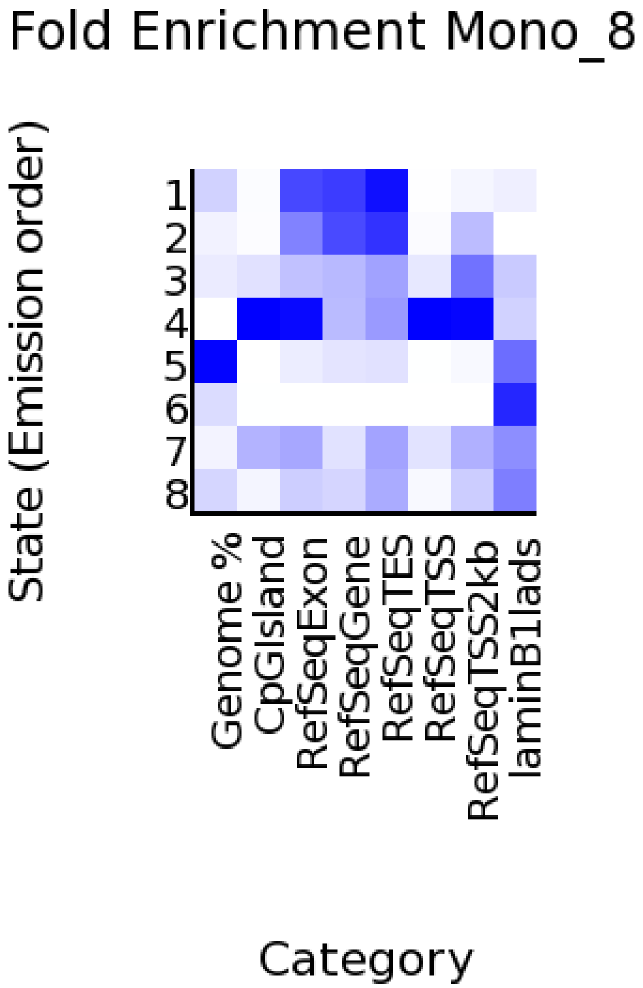

- Mono_8 Overlap Enrichment SVG File
- Mono_8 Overlap Enrichment Tab-Delimited Text File

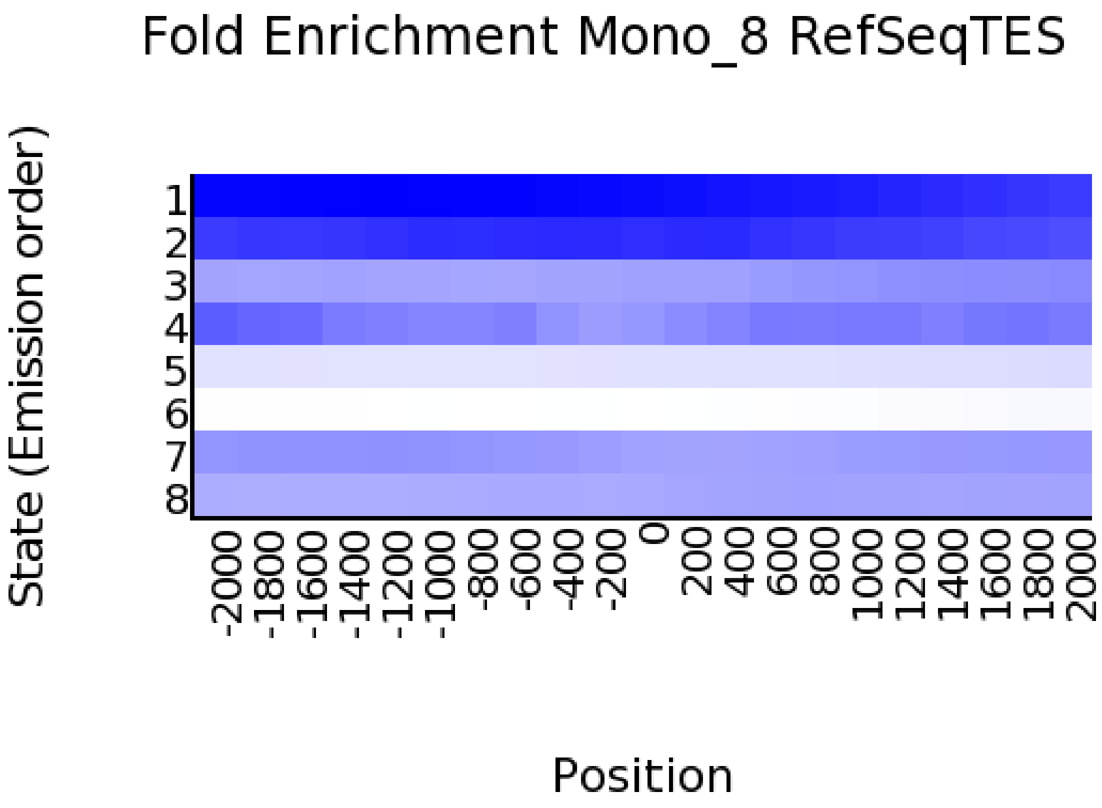

- Mono_8_RefSeqTES_neighborhood Enrichment SVG File
- Mono_8_RefSeqTES_neighborhood Enrichment Tab-Delimited Text File

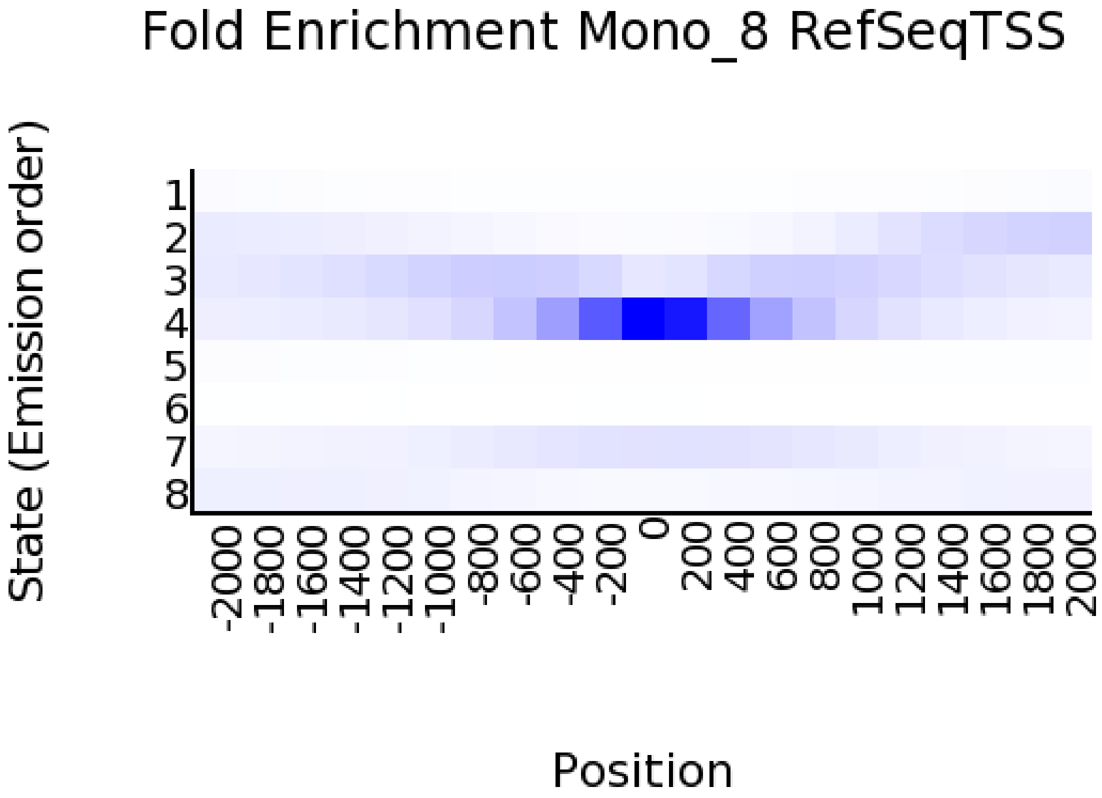

- Mono_8_RefSeqTSS_neighborhood Enrichment SVG File
- Mono_8_RefSeqTSS_neighborhood Enrichment Tab-Delimited Text File

Muscle_8 Enrichments

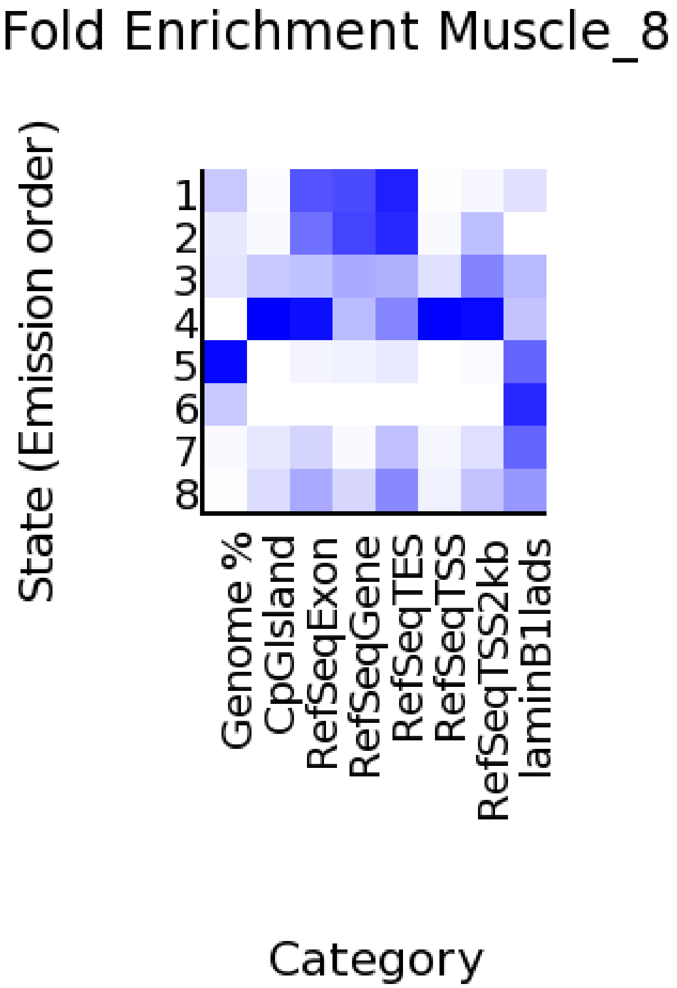

- Muscle_8 Overlap Enrichment SVG File
- Muscle_8 Overlap Enrichment Tab-Delimited Text File

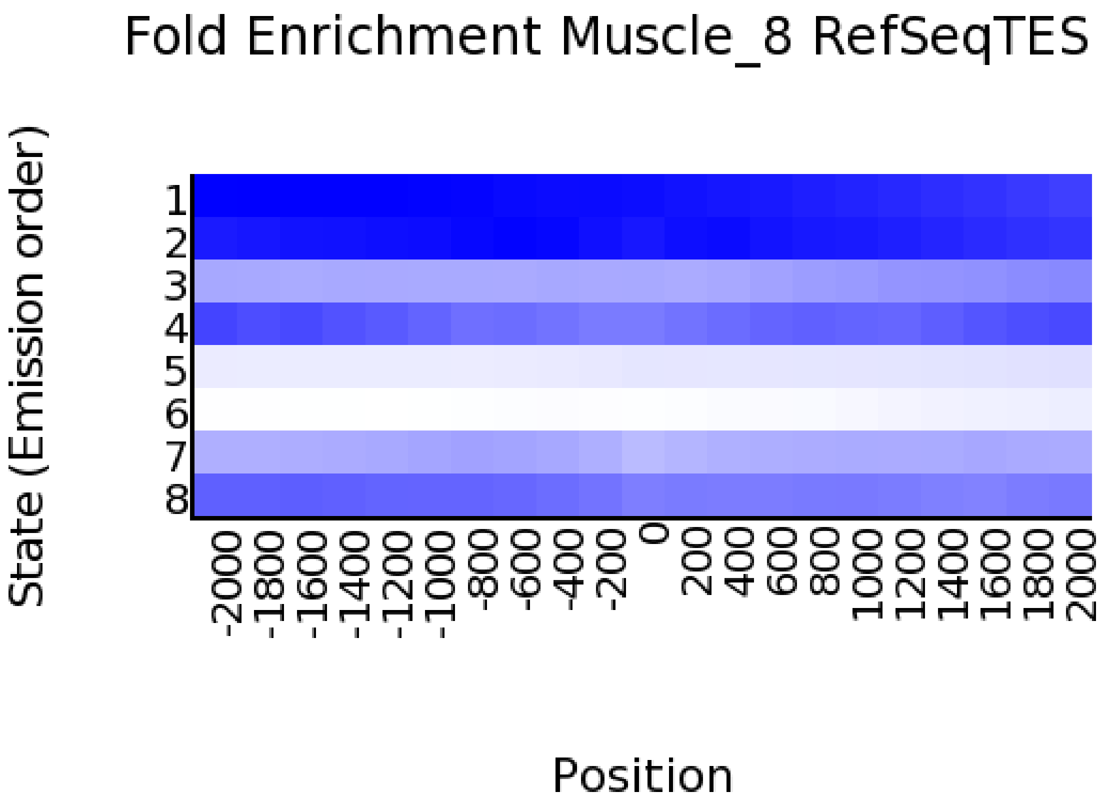

- Muscle_8_RefSeqTES_neighborhood Enrichment SVG File
- Muscle_8_RefSeqTES_neighborhood Enrichment Tab-Delimited Text File

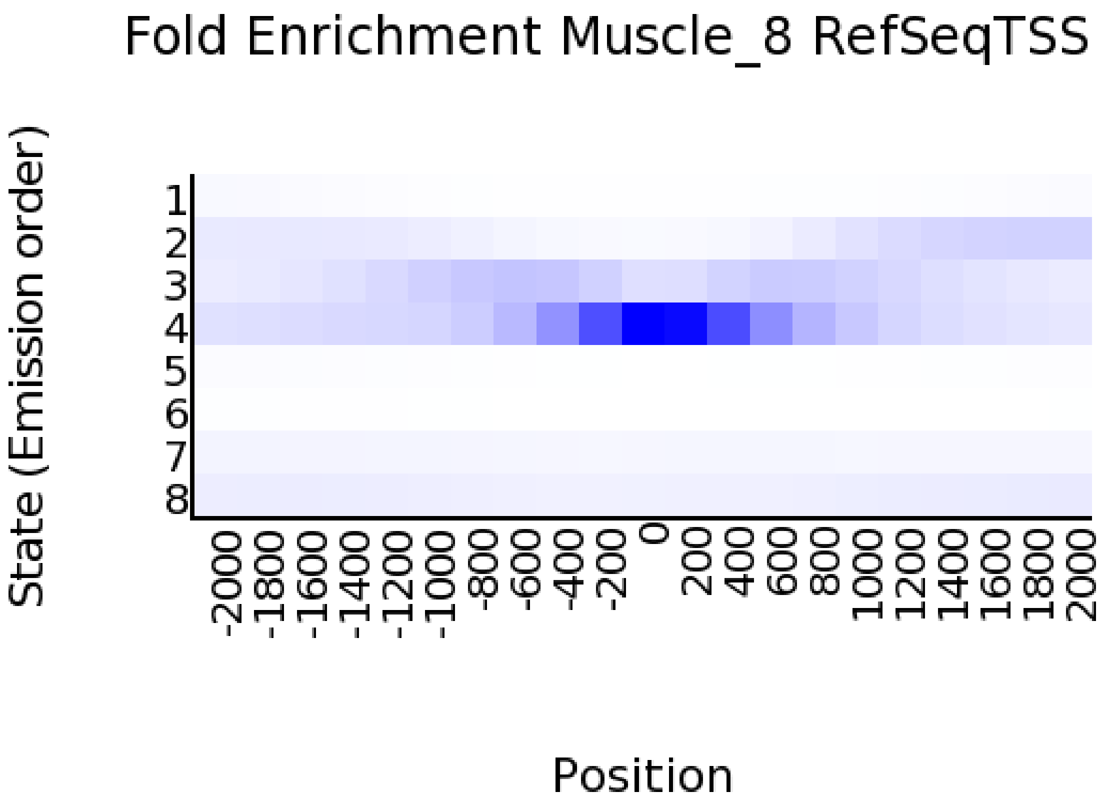

- Muscle_8_RefSeqTSS_neighborhood Enrichment SVG File
- Muscle_8_RefSeqTSS_neighborhood Enrichment Tab-Delimited Text File

TC_8 Enrichments

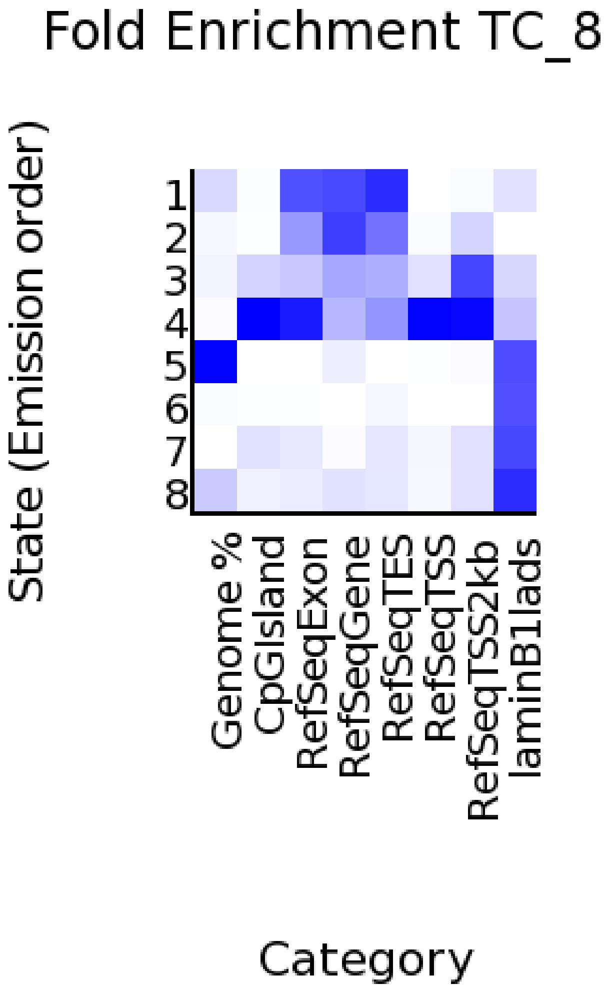

- TC_8 Overlap Enrichment SVG File
- TC_8 Overlap Enrichment Tab-Delimited Text File

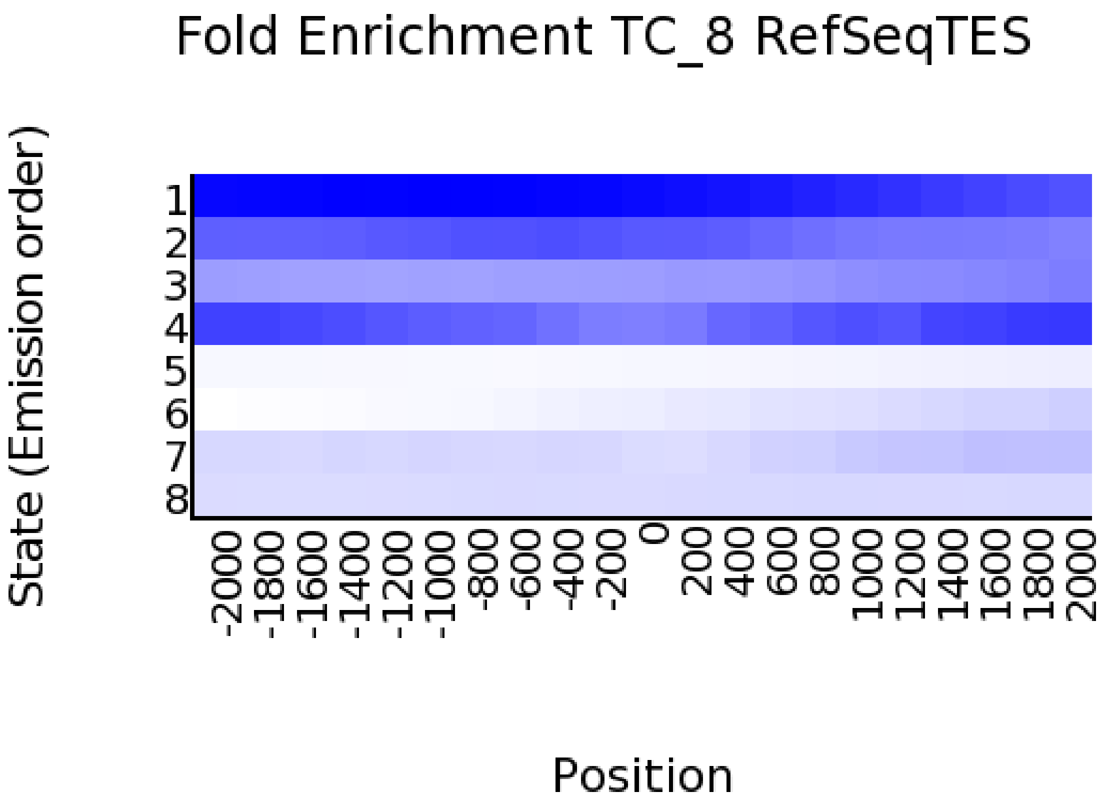

- TC_8_RefSeqTES_neighborhood Enrichment SVG File
- TC_8_RefSeqTES_neighborhood Enrichment Tab-Delimited Text File

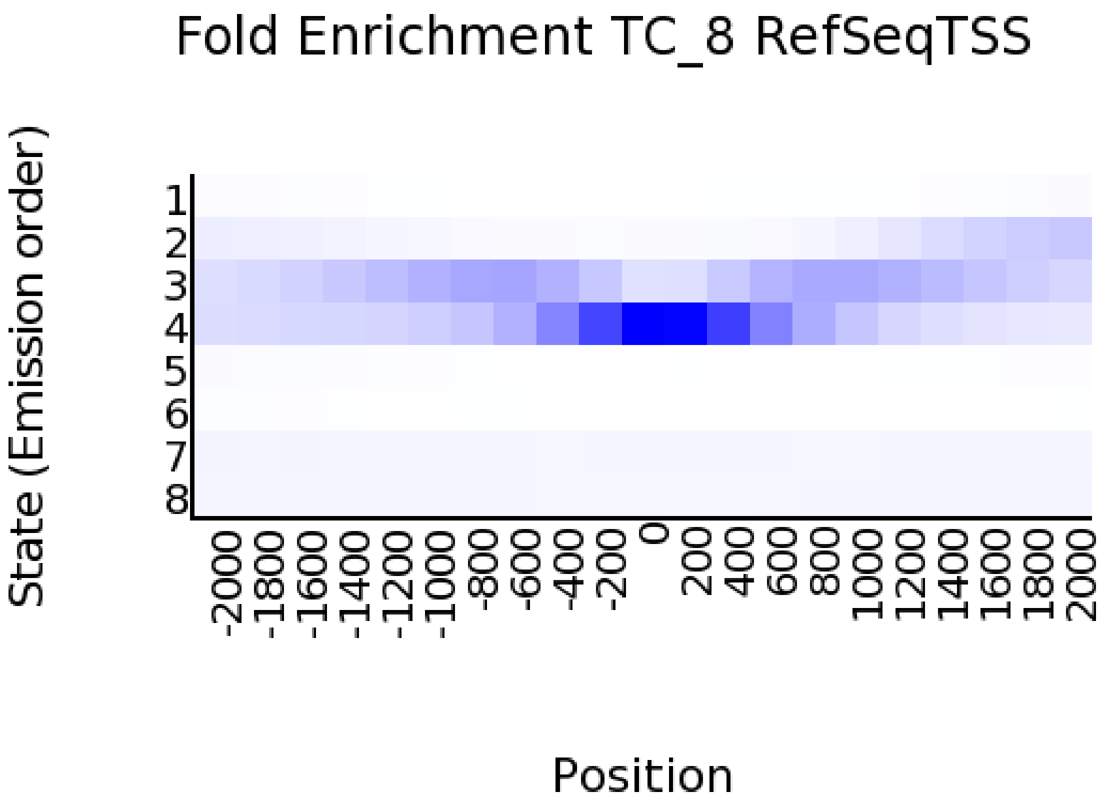

- TC_8_RefSeqTSS_neighborhood Enrichment SVG File
- TC_8_RefSeqTSS_neighborhood Enrichment Tab-Delimited Text File
